# Efficacy and Safety of Shenfu Injection for Severe Pneumonia in the Elderly: A Systematic Review and Meta-Analysis Based on Western and Eastern Medicine

**DOI:** 10.3389/fphar.2022.779942

**Published:** 2022-08-25

**Authors:** Shihua Shi, Fei Wang, Bonan Chen, Jie Pan, Dan Luo, Caixia Pei, Demei Huang, Xiaomin Wang, Yilan Wang, Zherui Shen, Weihao Li, Yongcan Wu, Yacong He, Zhenxing Wang

**Affiliations:** ^1^ Department of Geriatric, Hospital of Chengdu University of Traditional Chinese Medicine, Chengdu, China; ^2^ Department of Epidemiology and Public Health, Swiss Tropical and Public Health Institute, Basel, Switzerland; ^3^ Faculty of Science, University of Basel, Basel, Switzerland; ^4^ State Key Laboratory of Translational Oncology, Department of Anatomical and Cellular Pathology, Prince of Wales Hospital, The Chinese University of Hong Kong, Hong Kong, China; ^5^ Department of Pathology, Stanford University School of Medicine, Palo Alto, CA, United States; ^6^ Cardiology Division, West China Hospital, Sichuan University, Chengdu, China; ^7^ Chongqing Key Laboratory of Traditional Chinese Medicine for Prevention and Cure of Metabolic Diseases, Chongqing, China; ^8^ College of Traditional Chinese Medicine, Chongqing Medical University, Chongqing, China

**Keywords:** Shenfu injection, severe pneumonia, aged, biomarkers, COVID-19, mortality rate, meta-analysis

## Abstract

**Background:** Although increasing clinical trials studying Shenfu injection (SFI) comprising panaxoside 0.8 mg/ml extracted from Panax ginseng C.A. Mey. and aconitine 0.1 mg/ml extracted from *Aconitum carmichaeli* Debeaux for elderly patients with severe pneumonia on biomarkers associated with COVID-19 progression are emerging, there is no evidence-based evaluation for the effect of SFI on elderly severe pneumonia.

**Objectives:** To evaluate the effect of SFI on elderly patients with severe pneumonia providing hints for treating critical COVID-19, we conducted a systematic review and meta-analysis.

**Methods:** Nine databases, namely, PubMed, EMBASE, Web of Science, Science Direct, Google Scholar, Wanfang, Chongqing VIP Database, CNKI, and SinoMed were used to search clinical trials reporting the effect of SFI as an adjuvant for elderly severe pneumonia on outcomes of interest. Primary outcomes were total effective rate, Acute Physiology and Chronic Health Evaluation (APACHE) II score, mortality, and safety. Secondary outcomes were predictors associated with COVID-19 progression. Duplicated or irrelevant articles with unavailable data were excluded. Cochrane Collaboration’s tool was used to evaluate the risk of bias by two reviewers independently. All data were analyzed by Rev Man 5.4. Continuous variables were shown as weighted mean difference (WMD) or standard mean difference (SMD) with 95% confidence intervals (95% CI), whereas dichotomous data were calculated as the risk ratio (RR) with 95% CI.

**Results:** We included 20 studies with 1, 909 participants, and the pooled data showed that compared with standard control, SFI could improve the total effective rate (RR = 1.25, 95% CI = 1.14–1.37, and *n* = 689), APACHE II score (WMD = −2.95, 95% CI = −3.35, −2.56, and *n* = 809), and predictors associated with COVID-19 progression (brain natriuretic peptide, creatine kinase, stroke volume, cardiac output, left ventricular ejection fraction, cardiac index, sE-selectin, von Willebrand factor, activated partial thromboplastin time, platelet counts, D-Dimer, procalcitonin, and WBC count). SFI may reduce mortality (RR = 0.52, 95% CI = 0.37–0.73, and *n* = 429) and safety concerns (RR = 0.29, 95% CI = 0.17–0.51, and *n* = 150) for elderly severe pneumonia.

**Conclusion:** SFI as an adjuvant may improve the total effective rate, APACHE II score, gas exchange, and predictors associated with COVID-19 progression, reducing mortality and safety concerns for elderly patients with severe pneumonia.

## 1 Introduction

Advancing age was a prominent risk factor for the mortality of severe pneumonia, and the risk of mortality of severe pneumonia increased with aging (Guzik et al., 2020). The high rate of morbidity, adverse clinical outcomes, and mortality in the severe acute respiratory syndrome (SARS) (Chan et al., 2003), coronavirus disease 2019 (COVID-19), and other severe pneumonia patients was closely correlated with older age (Zhou et al., 2020). Although multiple treatment candidates are under development, specific drugs to treat the elderly with severe COVID-19 have not been available since the emergency pandemic began. Compared with younger adults, the elderly should be paid more attention due to impaired mucociliary clearance, a waning immune system, and underlying comorbid diseases (El-Solh et al., 2001). Therefore, it is essential to find a better treatment for elderly severe pneumonia, omitted in the previous clinical studies.

Recent research studies highlighted the role of biomarkers identified as markers for potential progression to a critical or fatal illness, namely, hematological, inflammatory, immunological, and biochemical, especially those associated with coagulation cascades in disseminated intravascular coagulation and acute respiratory distress syndrome (Hu et al., 2020; Ponti et al., 2020). Cytokine-mediated coagulation disorders and systemic vasculitis were the dominant actors of multi-organ failure in elderly patients with severe COVID-19 complications (El-Solh et al., 2001). Elderly patients with severe or fatal COVID-19 had markedly increased brain natriuretic peptide (BNP), creatine kinase (CK), sE-selectin, von Willebrand factor (vWF), activated partial thromboplastin time (APTT), D-Dimer, and procalcitonin (PCT) compared to non-severe disease and survivors (Henry et al., 2020). Analyzing the effect of treatments on biochemical, inflammatory, immunologic biomarkers, and endothelial perturbation in elderly patients with severe pneumonia is of high scientific significance since these profiles are associated with the progression of severe acute respiratory syndrome coronavirus 2 (SARS-CoV-2). It may give some hints on COVID-19 treatments.

While there is generally no direct and specific treatment for severe COVID-19 among elderly patients, some botanical drugs reported have clear potential as adjunctive therapies (Frost et al., 2021). With the rise of “WE” medicine (a melding of Western medicine focused on microscopic and single-disease targets and Eastern medicine, exemplified by traditional Chinese therapies), which was proposed by professor Yung-Chi Cheng from Yale University (Shi et al., 2021b), emerging clinical trials have reported that Shenfu injection (SFI), of which the composition is panaxoside 0.8 mg/ml and aconitine 0.1 mg/ml, has been considered beneficial for elderly patients with severe or fatal forms of pneumonia based on traditional Chinese medicine (TCM) and modern empirical knowledge (Lv et al., 2017; Liu, 2020a; Shi et al., 2021a). SFI was originated from Shenfu decoction consisting of Panax ginseng C.A. Mey. 15 g and Aconitum carmichaeli Debeaux 30 g, and recent research has reported that Shenfu decoction, with multicomponents and multitargets, may regulate immunity and apoptosis to treat severe COVID-19 patients (Li et al., 2020). In addition, recent studies have reported promising pharmacological activities of SFI in alleviating acute lung injury (Wang et al., 2008). SFI may block the vicious circle of the inflammatory response and improve immunity, and have anti-shock effects, offering a solution to the current health care challenge. However, the impact of SFI on severe pneumonia in elder patients remains poorly understood and has not been evaluated systematically and comprehensively. We thereby performed a systematic review and meta-analysis in older patients with severe pneumonia, specifically on biomarkers associated with COVID-19 progression, to provide evidence-based guideline recommendations according to results of clinical trials to help clinical decision-making in older patients with severe pneumonia, providing some hints on the treatments of severe COVID-19.

## 2 Methods

The present systematic review and meta-analysis was conducted based on the Preferred Reporting Items for Systematic Reviews and Meta-Analyses: The PRISMA Statement (Page et al., 2021). The study protocol was registered with the prospective register of systematic reviews (PROSPERO) database (CRD42021276939).

### 2.1 Search Strategies

The sources utilized to conduct this systematic review and meta-analysis were articles published in five English databases, namely, PubMed, EMBASE, Web of Science, Science Direct, and Google Scholar and four Chinese databases, namely, the Wanfang Database, Chongqing VIP Database, Chinese National Knowledge Infrastructure (CNKI), and SinoMed. To widen the search coverage, the International Clinical Trials Registry Platform and the Chinese Clinical Trial Registry were also utilized to identify unpublished trials in this context. Literature published up to 4 September 2021, with an updated search performed on 20 March 2022, was selected. The additional relevant trials identified from the references of systematic reviews and eligible studies were also retrieved. The comprehensive search strategy was conducted using two categories of keywords for PubMed, and the search terms were modified to suit other databases: (“Shenfu injection” OR “SFI” OR “Shenfu” OR “Shenfu decoction” OR “SF injection” OR “SF” OR “ginsenoside” OR “aconite total alkaloids” OR “Panax ginseng C. A. Meyer” OR “Radix Aconitum carmichaeli”) AND (“coronavirus disease 2019” OR “COVID-19” OR “severe acute respiratory syndrome coronavirus 2” OR “SARS-CoV-2” OR “coronavirus” OR “novel coronavirus” OR “nCoV” OR “2019-nCoV” OR “severe pneumonia” OR “Severe Acute Respiratory Syndrome”). The Chinese databases were also searched by the abovementioned search terms, translated into Chinese. Search strategies for selecting the keyword, title, or abstract fields were different, referring to the specific databases. There were no language, date, or publication status limitations for the inclusion of eligible studies to reduce the risk of publication bias. Detailed search strategies were presented in the additional file.

### 2.2 Selection Criteria

The study screen and the selection process were performed independently by two investigators (SS and DL). After removing duplicate articles, the titles, abstracts, and full texts were screened, applying the selection criteria to select potentially eligible articles. The differences in data screening and selection were resolved through panel discussions or settled by a third reviewer (FW) to resolve doubt or disagreement. Given that randomized controlled trials (RCTs) on the optimal treatments for older patients with severe COVID-19 were lacking and clinical trials investigating SFI for severe pneumonia including COVID-19 may be limited, RCTs and quasi RCTs were both considered to be included in our study, due to the urgent nature of the COVID-19 pandemic. Finally, RCTs or quasi RCTs studying the effects of SFI on the elderly with severe pneumonia were included. Table 1 presented the Participants, Intervention, Comparators, Outcomes, and Study Design (PICOS) criteria.

**TABLE 1:** Participants, intervention, comparators, outcomes, and study design (PICOS).

Parameters	Descriptions
Populations	Elderly patients with severe pneumonia
Interventions	Shenfu injection
Comparators	Standard control
Outcomes	The total effective rate, the Acute Physiology and Chronic Health Evaluation (APACHE) II score, mortality rate, predictors associated with COVID-19 disease progression, and safety
Study designs	Randomized controlled trials (RCT) or quasi RCTs

The primary outcome measures of interest were the total effective rate and the Acute Physiology and Chronic Health Evaluation (APACHE) II score predicting disease severity, mortality, and safety profiles. The clinical efficacy was defined based on Infectious Diseases Society of America/American Thoracic Society Consensus Guidelines on the Management of Community-Acquired Pneumonia in Adults (Mandell et al., 2007); markedly effective: the clinical symptoms such as fever, cough, and chest tightness were significantly alleviated, and all vital indicators returned to normal levels; effective: clinical symptoms were relieved, and most indicators returned to normal levels; the total effective rate = [(number of markedly effective cases + number of effective cases)/number of cases] × 100%. Safety concerns mainly included dyspnea, nausea, vomiting, adverse cardiovascular events (arrhythmia, unstable angina, abnormal blood pressure, and myocardial infarction), and coma that occurred in severe pneumonia. In addition, predictors associated with COVID-19 disease progression, namely, BNP, CK, stroke volume (SV), cardiac output (CO), cardiac index (CI, CO indexed to body surface area), left ventricular ejection fraction (LVEF), soluble endothelial selectin (sE-selectin), APTT, vWF, D-Dimer, platelet counts, C-reactive protein (CRP), white blood cell (WBC) count, PCT, partial pressure of arterial oxygen (PaO_2_), and lactic acid accumulation were the secondary outcome measures of interest.

Included studies were supposed to meet inclusion criteria as follows: 1) RCTs or quasi RCTs including prospective cohort studies; 2) elderly patients: the trials reported that the patients included were elderly patients with average age ≥ 60; 3) patients diagnosed with severe pneumonia with one of the vital criteria (septic shock or mechanical ventilation) or at least three of the secondary criteria including arterial oxygen pressure/fraction of inspired oxygen (PaO_2_/FiO2) ratio < 250 mmHg, respiratory rate > 30 breaths/min, WBC count < 4 × 10^9^, blood urea nitrogen level ≥ 30 mg/dl, multilobar infiltrates, platelet count < 10 × 10^9^, confusion, hypotension requiring aggressive fluid resuscitation, or core temperature < 36°C (Mandell et al., 2007); 4) comparison of SFI as an adjuvant with standard control alone; SFI could be delivered *via* an intravenous drip, intravenous injection, or pumps; 5) trials that investigated at least one of the outcomes of interest.

Exclusion criteria were as follows: duplicated studies; articles with unavailable data; retrospective cohort studies, crossover trials, cluster-randomized control studies, and the RCTs with factorial design or sequential design; the effects of SFI as an adjuvant could not be evaluated because of the involvement of other TCM treatments.

### 2.3 The Extraction and Management of Data

For each included article, two scholars (SS and ZW) independently and simultaneously collected information on participant characteristics, study procedure, and primary outcomes using a standardized, pilot-tested data collection form and then crosschecked. The following items were collected: 1) first author’s name and publication date; 2) study location; 3) sample size; 4) subject characteristics (patient age, gender); 5) intervening measure and the source; 6) dose of SFI; 7) treatment duration; 8) outcome measures. A third researcher (FW) checked the collected data and resolved any discrepancies.

### 2.4 Quality Appraisal

The risk of bias in the included studies through Cochrane Collaboration’s tool was independently assessed by two reviewers (SS and BC) in the following seven aspects: random sequence generation, allocation concealment, blinding of participants and personnel, blinding of outcome assessment, incomplete outcome data, selective reporting, and other bias (Higgins et al., 2011). A third reviewer (WL) independently repeated the data quality assessment to settle the disagreements when necessary. The statistical analyses were conducted using RevMan 5.4 (The Nordic Cochrane Centre, The Cochrane Collaboration, Copenhagen).

### 2.5 Data Synthesis

To analyze the effect size for each outcome parameter, continuous variables were shown as weighted mean difference (WMD) with 95% confidence intervals (95% CI), whereas dichotomous data were calculated as the risk ratio (RR) with 95% CI. The data utilized the standard mean difference (SMD) to reduce the difference, if there was a difference in the measurement unit (Riley et al., 2011). The random-effect model or fixed-effect model was adopted to analyze the pooled statistics. The selection of the effect model was according to the heterogeneity test: the random-effect model was utilized if statistical heterogeneity was identified (I^2^ > 50% or *p* < 0.1); conversely, a fixed-effect model was adopted in the absence of substantial heterogeneity (I^2^ ≤ 50% and *p* ≥ 0.1) (Higgins et al., 2003). *p* values < 0.05 were identified to be statistically significant. Subgroup analysis was performed based on the study design. Sensitivity analysis was carried out to identify possible outlier studies with a high risk of bias or industry funding, which may affect our results by excluding one study each time from the pooled analysis. Publication bias was measured by the Eggers test and analyzed using funnel plots when ≥ 10 studies were included (Egger et al., 1997; Sterne and Egger, 2001). Rev Man 5.4. was used to conduct the data analysis. The certainty of the evidence was assessed by GRADEprofiler (version 3.6).

## 3 Results

### 3.1 Flow and Characteristics of the Selected Studies

**FIGURE 1:**
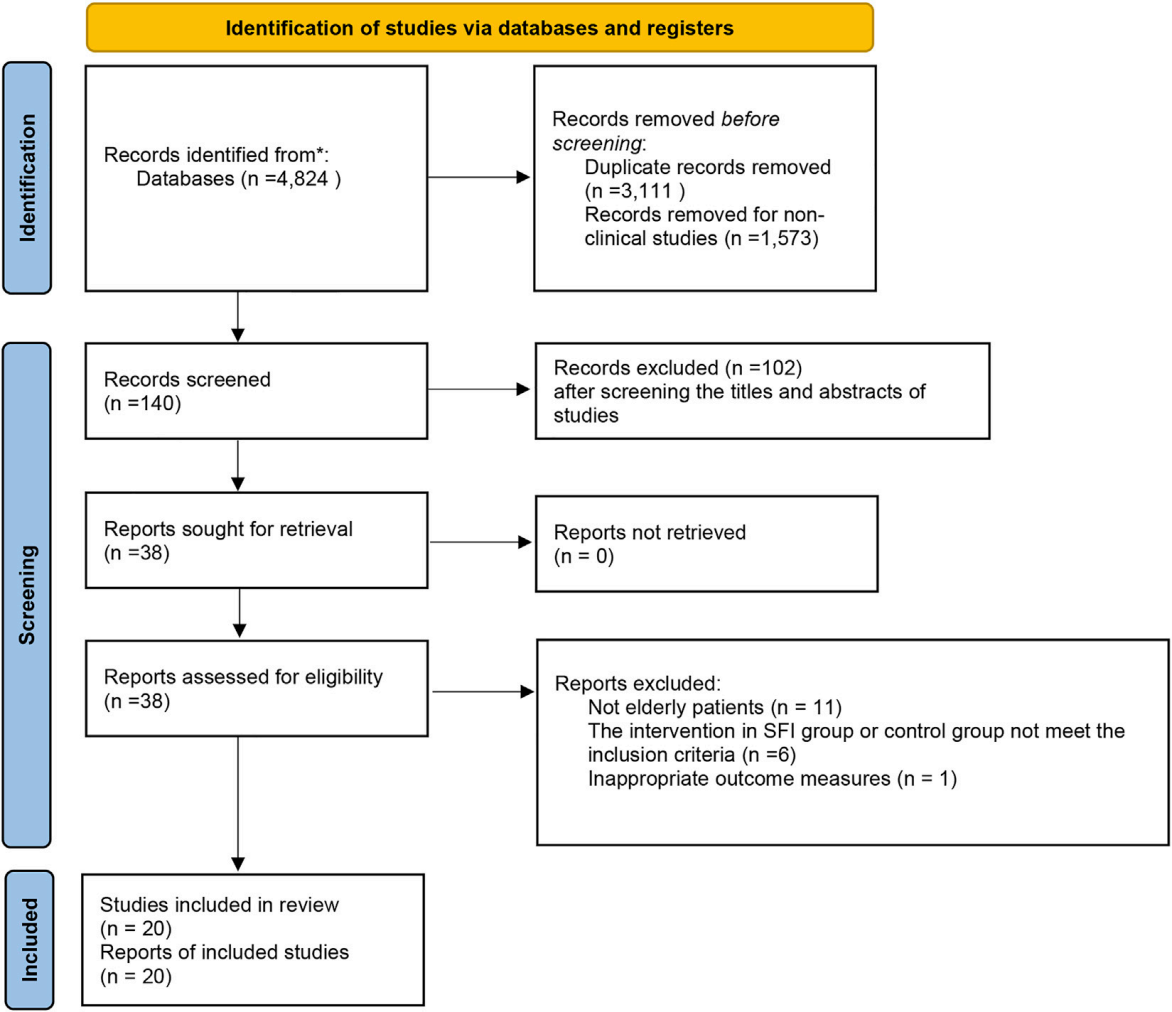
Flow diagram for the process of included study identification.

After an initial search from the five English and four Chinese databases was performed, 4,824 articles were identified. Of these, 3,111 articles were eliminated due to duplicate collections. About 1,573 publications were further precluded since they were non-clinical studies. After screening the titles and abstracts of studies for potential trials, a total of 38 possibly relevant studies were obtained for full-text screening. Then, 18 articles were eliminated for the following reasons: not elderly patients, the intervention in the SFI group or control group did not meet the inclusion criteria, or have inappropriate outcome measures (Additional file). Finally, 20 clinical trials published from 2013 to 2022 were eligible (Figure 1).

The included studies were published in Chinese or English, with a total of 1,909 patients. There were 977 patients in the experimental group and 932 patients in the control group among these 20 clinical trials. Patients were divided into the SFI group (SFI plus standard control) and control group (standard control) with no significant difference between these two groups in general information, such as age, gender, body temperature, respiratory rate, heart rate, the inflammatory mediator, course of the disease, or comorbidities. The SFI, prepared according to the Chinese pharmacopeia, was obtained from Ya’an Sanjiu Pharmaceutical Co., Ltd. or Shenzhen Huarun Sanjiu Pharmaceutical Trade Co. Ltd., with chemical analysis based on previous high-performance liquid chromatography (HPLC) research (Table 2), and no studies were funded by the industry.

**TABLE 2:** Summary of the eligible studies included in the meta-analysis.

Study (author/year)	Study design	Location	Trial				Control				SFI dose	Source	Composition	Quality control	Chemical analysis	Duration	Data selected
			N	Male	Female	Age	N	Male	Female	Age			(Species, concentration)				
Chen et al. (2016)	RCT	Guangdong, Guangzhou Province	60	28	32	68.3 ± 2.5	60	30	30	67.5 ± 4.1	60 ml	SFDA: Z51020664, Ya ‘an Sanjiu Pharmaceutical Co., Ltd.	Panax ginseng C. A. Mey 0.1 g/ml, Aconitum carmichaelii Debeaux 0.2 g/ml	Prepared according to Chinese pharmacopeia	HPLC	Based on the patient’s condition	b, c, e, f, g, h, k
Dong et al. (2017)	RCT	Cangshan, Shandong Province	60	NR	NR	66.85 ± 8.42	60	NR	NR	66.85 ± 8.42	100 ml, bid	NR	NR	NR	NR	7 days	b, i, l, m
Li et al. (2015a)	RCT	Tangshan, Hebei Province	40	18	22	69.1 ± 2.7	40	20	20	68.2 ± 3.4	60 ml	NR	NR	NR	NR	Based on the patient’s condition	b, c, e, f, g, h, j
Li et al. (2015b)	RCT	Tangshan, Hebei Province	42	NR	NR	NR	42	NR	NR	NR	60 ml	NR	NR	NR	NR	Based on the patient’s condition	a, b, c, n, r
Li et al. (2019)	RCT	Changzhou, Jiangsu Province	35	20	15	75.3 ± 9.2	35	22	13	75.5 ± 8.1	60 ml	SFDA: Z51020664, Ya ‘an Sanjiu Pharmaceutical Co., Ltd.	Panax ginseng C. A. Mey 0.1 g/ml, Aconitum carmichaelii Debeaux 0.2 g/ml	Prepared according to Chinese pharmacopeia	HPLC	7 days	d, I, j, p, r
Li (2019)	RCT	Beijing	52	30	22	67.93 ± 5.29	52	31	21	66.89 ± 5.1	100 ml, qd	SFDA: Z20043116, Ya ‘an Sanjiu Pharmaceutical Co., Ltd.	Panax ginseng C. A. Mey 0.1 g/ml, Aconitum carmichaelii Debeaux 0.2 g/ml	Prepared according to Chinese pharmacopeia	HPLC	14 days	b, I, j, k, l, m
Lin et al. (2013)	RCT	Guangzhou	33	19	14	79.8 ± 12.60	16	10	6	76.5 ± 13.2	60 ml, bid	Ya’an Sanjiu Pharmaceutical Co., Ltd.	Panax ginseng C. A. Mey 0.1 g/ml, Aconitum carmichaelii Debeaux 0.2 g/ml	Prepared according to Chinese pharmacopeia	HPLC	Based on the patient’s condition	q, r
Liu, (2020a)	RCT	Beijing	40	23	17	67.5 ± 3.2	40	22	18	67.5 ± 3.0	100 ml	SFDA: Z20043116, Ya ‘an Sanjiu Pharmaceutical Co., Ltd.	Panax ginseng C. A. Mey 0.1 g/ml, Aconitum carmichaelii Debeaux 0.2 g/ml	Prepared according to Chinese pharmacopeia	HPLC	Based on the patient’s condition	a
Lv et al. (2017)	RCT	Hangzhou	45	26	19	67.2 ± 15.0	44	25	19	65.4 ± 6.7	50 ml, bid	SFDA: Z20043117, Shenzhen Huarun Sanjiu Pharmaceutical Trade Co. Ltd.	Panax ginseng C. A. Mey 0.1 g/ml, Aconitum carmichaelii Debeaux 0.2 g/ml	Prepared according to Chinese pharmacopeia	HPLC	14 days	b, o, p
Qu and Shao, (2018)	RCT	Xinjiang	30	16	14	63.12 ± 2.59	30	17	13	62.19 ± 3.06	60 ml	NR	NR	NR	NR	Based on the patient’s condition	b, c, n,
Shi et al. (2021a)	RCT	Zhengzhou, Henan Province	15	8	7	73.7 ± 6.3	15	9	6	74.2 ± 6.5	50 ml, bid	NR	NR	NR	NR	Based on the patient’s condition	a, p, q, s
Sun et al. (2018)	RCT	Hebei	42	27	15	61.72 ± 11.43	42	28	14	62.71 ± 12.45	60 ml	SFDA: Z20043117	Panax ginseng C. A. Mey 0.1 g/ml, Aconitum carmichaelii Debeaux 0.2 g/ml	Prepared according to Chinese pharmacopeia	HPLC	7 days	a, d, e, f, g, h
Wang et al. (2017)	RCT	Hebei	89	NR	NR	67.9 ± 7.6	87	NR	NR	67.9 ± 7.6	100 ml, bid	NR	NR	NR	NR	7 days	b, i, j, k, l, m
Wang (2020)	Quasi RCT	Xian	39	16	23	71.45 ± 5.26	39	17	22	72.06 ± 5.1	50 ml, qd	NR	NR	NR	NR	7 days	a, n, o
Zheng et al. (2020)	Quasi RCT	Henan	41	22	19	69.23 ± 4.25	41	23	18	68.27 ± 4.12	100 ml, qd	SFDA: Z20043116	Panax ginseng C. A. Mey 0.1 g/ml, Aconitum carmichaelii Debeaux 0.2 g/ml	Prepared according to Chinese pharmacopeia	HPLC	7 days	b, c, k, n
Zhi et al. (2014)	RCT	Ningbo	21	14	7	61–83	21	12	9	63–86	50 ml, qd	Ya’an Sanjiu Pharmaceutical Co., Ltd.	Panax ginseng C. A. Mey 0.1 g/ml, Aconitum carmichaelii Debeaux 0.2 g/ml	Prepared according to Chinese pharmacopeia	HPLC	7 days	p, r
Ren, (2020)	Quasi RCT	Nanyang, Henan Province	60	25	35	69.66 ± 3.38	60	27	33	68.36 ± 3.19	60 ml	SFDA: Z51020664, Huarun Sanjiu (Ya’an)Pharmaceutical Trade Co. Ltd.	Panax ginseng C. A. Mey 0.1 g/ml, Aconitum carmichaelii Debeaux 0.2 g/ml	Prepared according to Chinese pharmacopeia	HPLC	7 days	a, c, i, s
Wang et al. (2015)	Quasi RCT	Zunhua, Hebei Province	98	NR	NR	NR	86	NR	NR	NR	NR	NR	NR	NR	NR	10 days	a, e, f, g, h, r
Kong et al. (2022)	RCT	Guangdong, Guangzhou Province	37	20	17	74.83 ± 4.61	36	19	17	75.61 ± 4.53	50 ml, qd	SFDA: Z51020664, Huarun Sanjiu Pharmaceutical Trade Co. Ltd.	Panax ginseng C. A. Mey 0.1 g/ml, Aconitum carmichaelii Debeaux 0.2 g/ml	Prepared according to Chinese pharmacopeia	HPLC	14 days	a, n, o, t

Note: bid, bis in die; qd, quaque die; Administration: a total effective rate; b, APACHE II score; c, BNP; d, creatine kinase (CK); e, LVEF; f, stroke volume (SV); g, cardiac output (CO); h, cardiac index (CI); HPLC, high-performance liquid chromatography; i, soluble endothelial selectin (sE-selectin); j, von Willebrand factor (vWF); k, activated partial thromboplastin time (APTT); l, platelet counts; m, D-Dimer; n, procalcitonin (PCT); NR, not report; o, C-reactive protein (CRP); p, PaO_2_; q, lactic acid accumulation; RCT, randomized controlled trial; r, mortality rate; s, safety profiles; SFDA, China state food, and drug; t, white blood counts.

### 3.2 Risk of Bias Assessment

**FIGURE 2:**
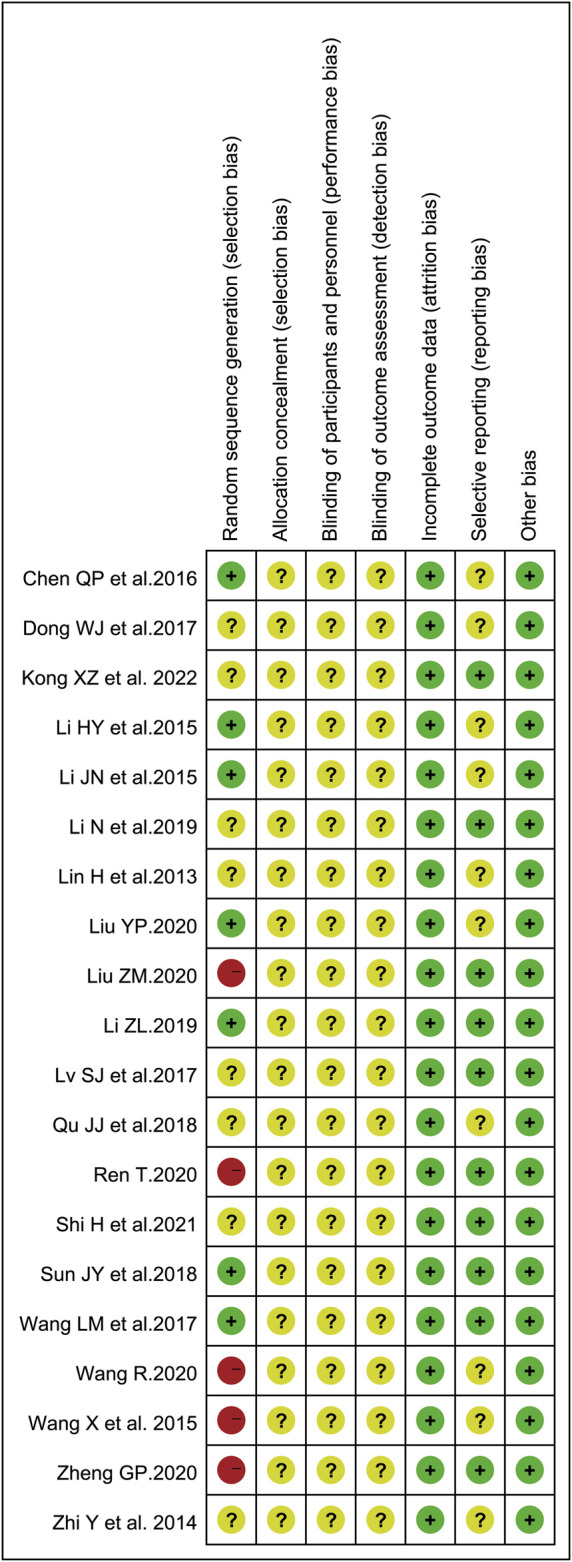
Risk of bias summary.

To evaluate the risk of bias in the involved studies, Cochrane Collaboration’s risk of bias assessment tool was utilized and the results were shown in Figure 2. There were seven trials describing the detailed stochastic methods used, and all used completely random number tables to generate the allocation sequence (Li et al., 2015a; Li et al., 2015b; Chen et al., 2016; Wang et al., 2017; Sun et al., 2018; Li, 2019; Liu, 2020a); while eight studies mentioned random sequence generation without the specific random method (Lin et al., 2013; Zhi et al., 2014; Dong et al., 2017; Lv et al., 2017; Qu and Shao, 2018; Li et al., 2019; Shi et al., 2021a; Kong et al., 2022). The blinding of subjects, researchers, or outcome assessment was not reported in all eligible articles. Whether prior protocols were designed was not reported in most trials. All involved articles showed a low risk of bias on incomplete outcome data.

### 3.3 Primary Outcomes

#### 3.3.1 The Total Effective Rate

**FIGURE 3:**
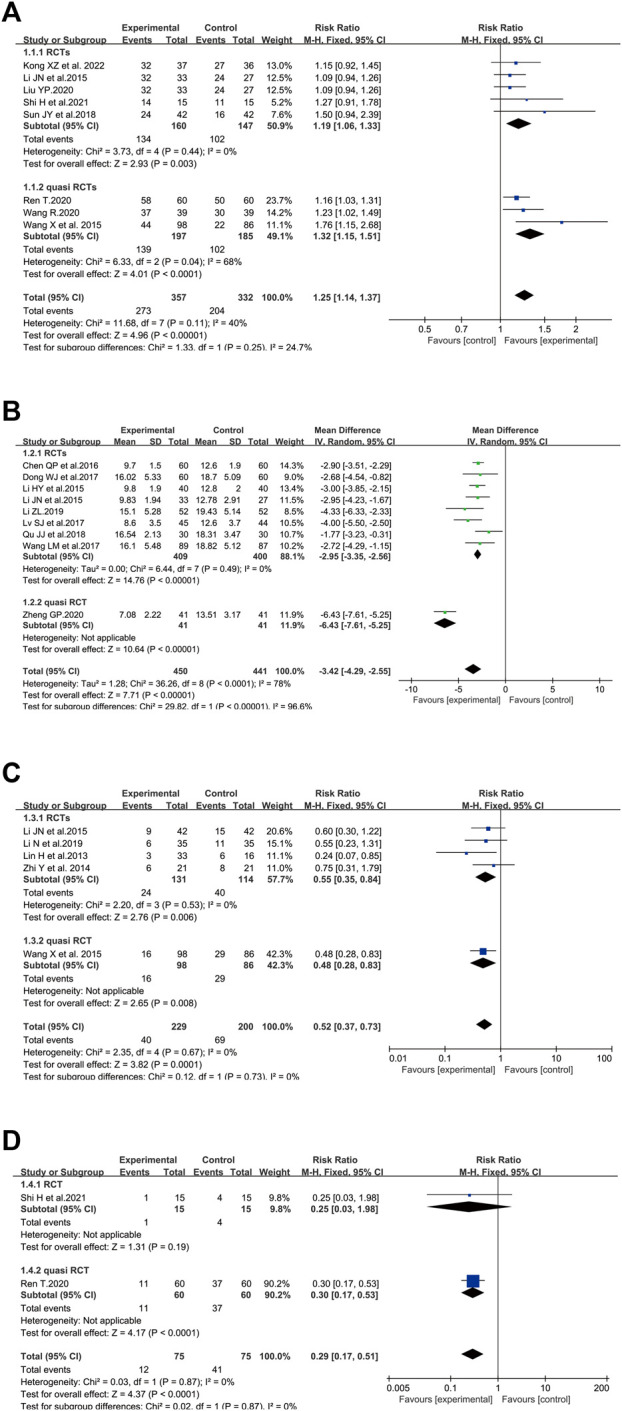
Meta-analysis results of SFI on primary outcomes: **(A)** total effective rate; **(B)** APACHE II score; **(C)** mortality rate; **(D)** safety concern.

A total of eight clinical trials involving 689 cases compared the total effective rate between the SFI group and the standard control group (Li et al., 2015b; Wang et al., 2015; Sun et al., 2018; Liu, 2020a; Ren, 2020; Wang, 2020; Shi et al., 2021a; Kong et al., 2022). In the present analysis, the pooled analysis revealed that elderly patients with severe pneumonia who underwent SFI plus standard control had a significantly improved total effective rate (RR = 1.25, 95% CI = 1.14–1.37, *p* < 0.00001) compared with the standard control alone. A fixed-effect model was utilized to carry out the meta-analysis since heterogeneity was not significant (*p* = 0.11, I^2^ = 40%). Subgroup analysis was performed based on the study design. The *p-value* of the test for subgroup differences was 0.25, and there was no significant heterogeneity between RCTs and quasi RCTs (Figure 3A).

#### 3.3.2 Acute Physiology and Chronic Health Evaluation II Score

There were nine studies involving 891 patients that measured the APACHE II score (Li et al., 2015a; Li et al., 2015b; Chen et al., 2016; Dong et al., 2017; Lv et al., 2017; Wang et al., 2017; Qu and Shao, 2018; Li, 2019; Zheng, 2020). The pooled results revealed that SFI plus standard control could significantly reduce the APACHE II score (WMD = −3.42, 95% CI = −4.29, −2.55) compared with the standard control alone. Statistical heterogeneity was identified in APACHE II score based on the heterogeneity test (*p* < 0.00001, I^2^ = 78%, *p* < 0.00001), and the random-effects model was adopted. The I^2^ of the test for subgroup differences was 96.6%, showing that the study design was the source of heterogeneity. The heterogeneity issue was resolved by subgroup analysis based on the study design. According to the subgroup of RCTs with homogeneity, SFI plus standard control could significantly reduce the APACHE II score (WMD = −2.95, 95% CI = −3.35, −2.56, *n* = 809) (Figure 3B).

#### 3.3.3 Mortality Rate

In terms of mortality rate, five trials with 429 participants measured the mortality rate (Lin et al., 2013; Zhi et al., 2014; Li et al., 2015b; Wang et al., 2015; Li et al., 2019) (Figure 3C). The results showed that the mortality rate in patients who adopted SFI plus standard control was decreased compared to participants treated by standard control alone (RR = 0.52, 95% CI = 0.37–0.73, *p* = 0.0001). No heterogeneity among the studies was found (*p* = 0.67, I^2^ = 0%), so a fixed-effect model was utilized to analyze the RR.

#### 3.3.4 Safety

A total of two trials involving 150 elderly severe pneumonia patients evaluated negative symptoms associated with conventional treatment or the progression of the disease (Ren, 2020; Shi et al., 2021a). Dyspnea, nausea, vomiting, arrhythmia, and coma were reported in Shi’s study (Shi et al., 2021a); complications, palindromia, and adverse cardiovascular events were reported in Ren’s study (Ren, 2020). As shown in Figure 3D, the safety concern of elderly patients in the combined group was dramatically less than that of the control group (RR = 0.29, 95% CI = 0.17–0.51, *p* < 0.0001). A *p*-value = 0.87 and I^2^ = 0% revealed that there was no heterogeneity among the studies. Therefore, a fixed-effect model was adopted to conduct the meta-analysis.

### 3.4 Secondary Outcomes (Predictors Associated With COVID-19 Disease Progression)

#### 3.4.1 Gas Exchange

##### 3.4.1.1 Partial Pressure of Arterial Oxygen

**FIGURE 4:**
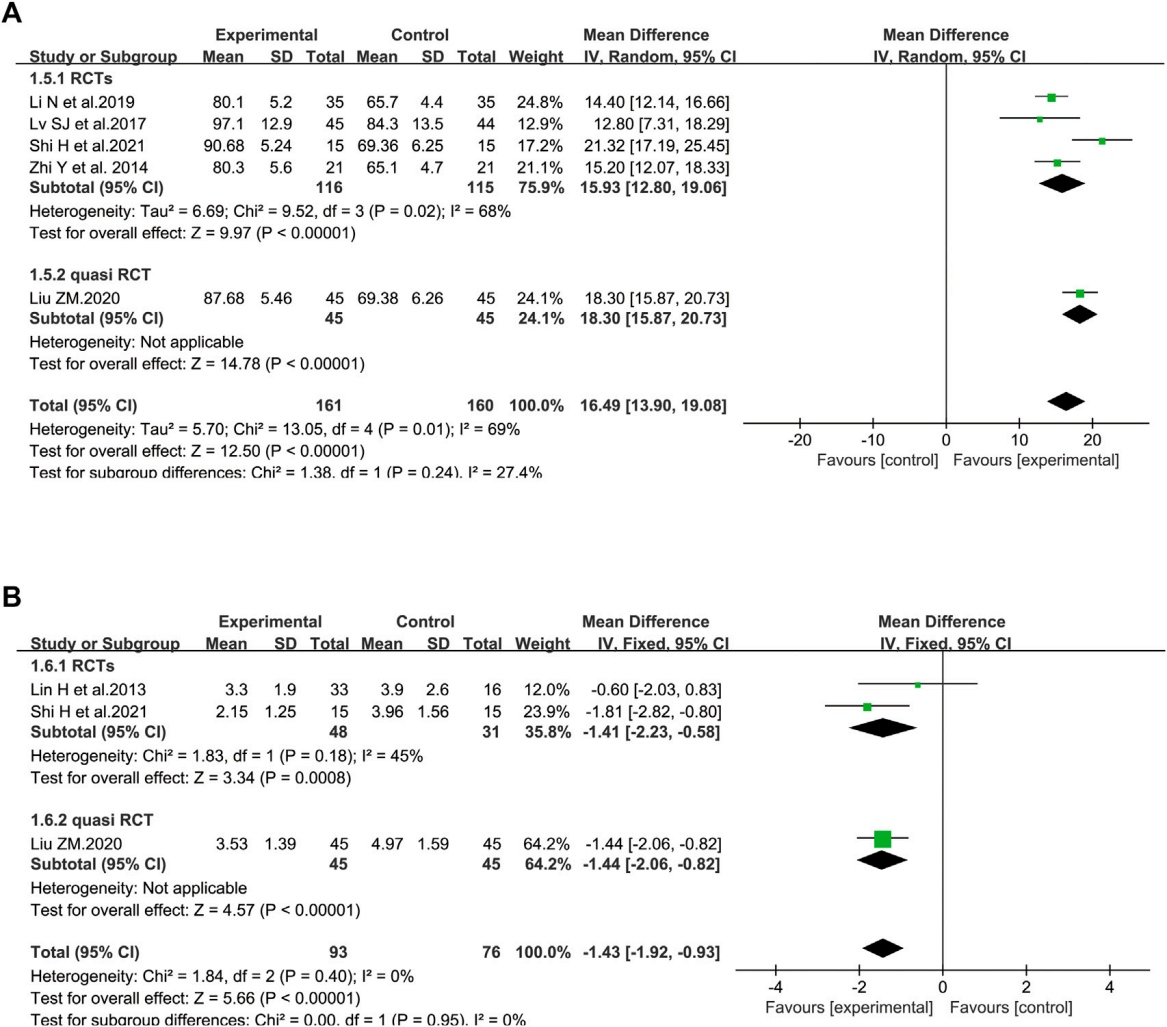
Meta-analysis results of gas exchange based on: **(A)** PaO_2_; **(B)** lactic acid accumulation.

In terms of PaO_2,_ five trials involving 321 patients were included (Zhi et al., 2014; Lv et al., 2017; Li et al., 2019; Liu, 2020b; Shi et al., 2021a). Considerable heterogeneity between these two studies was identified (I^2^ = 69%, *p* = 0.01). According to further analysis, the heterogeneity may be caused by two studies (Liu, 2020a; Shi et al., 2021a) since the I^2^ value was low to zero when these studies were removed. A random-effect model was utilized to perform the meta-analysis. SFI plus standard control achieved a greater improvement on PaO_2_ than the standard control (WMD = 16.49, 95% CI: 13.90–19.08, *p* < 0.00001), suggesting that SFI might increase the gas exchange for elderly severe pneumonia patients. Similar results were found in subgroup analysis based on the study design (Figure 4A).

##### 3.4.1.2 Lactic Acid Accumulation

The data about lactic acid accumulation were provided in three clinical studies (Lin et al., 2013; Liu, 2020b; Shi et al., 2021a). A fixed-effect model was used (Figure 4B) because data heterogeneity was not significant (I^2^ = 0%, *p* = 0.40). The pooled results revealed that the decrease in lactic acid accumulation levels was greater in the SFI group than that in the control group [WMD = −1.43, 95% CI: (−1.92, −0.93), *p* < 0.00001, *n* = 169].

#### 3.4.2 Inflammatory Markers

##### 3.4.2.1 Procalcitonin

**FIGURE 5:**
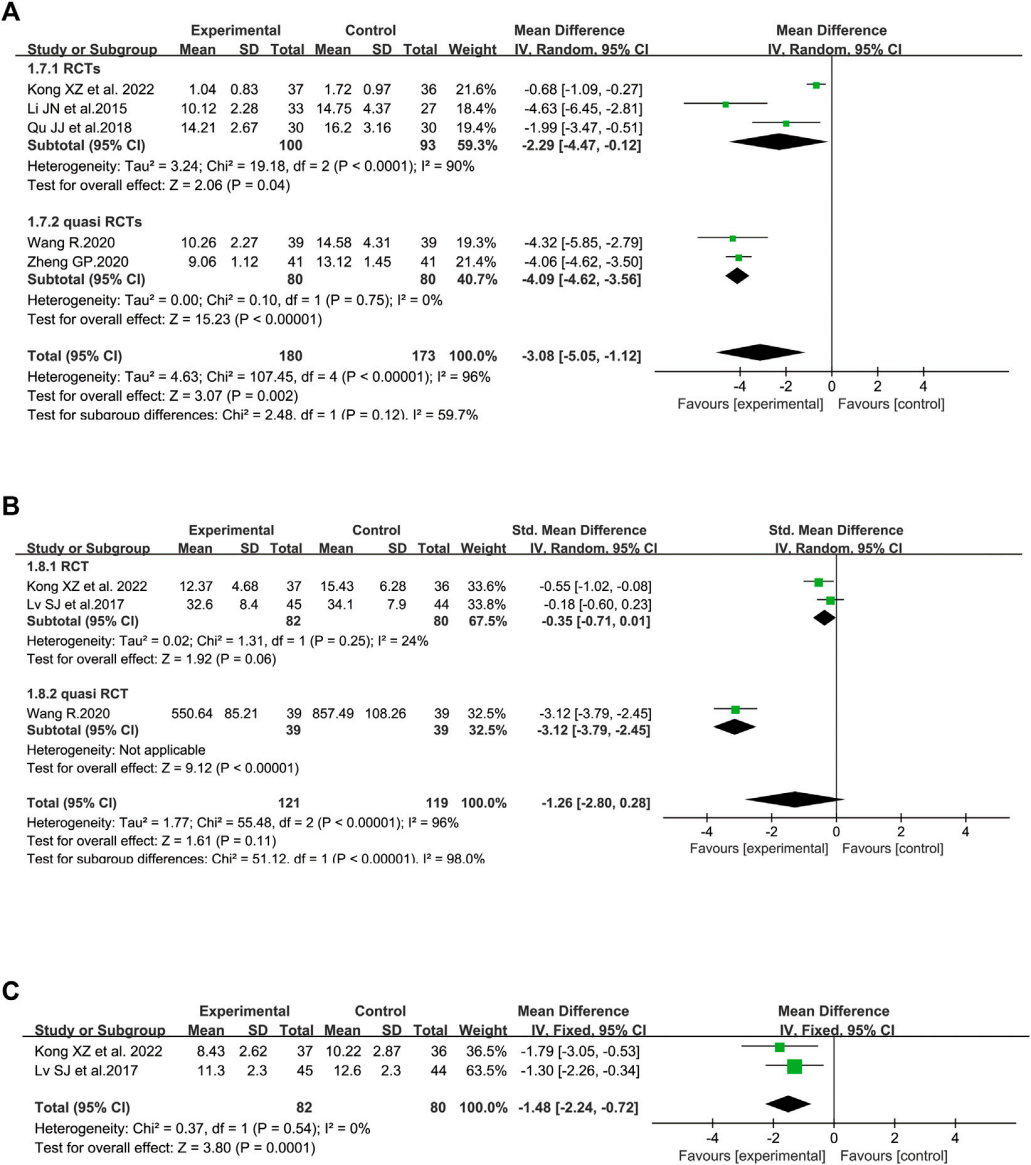
Meta-analysis results of inflammatory markers based on: **(A)** procalcitonin (PCT); **(B)** C-reactive protein (CRP); **(C)** white blood cell (WBC) counts.

Figure 5A illustrated the efficacy of SFI on PCT (Li et al., 2015b; Qu and Shao, 2018; Wang, 2020; Zheng, 2020; Kong et al., 2022) with 353 patients (173 in the control group and 180 in the SFI group). Compared with the control group, the decrease in PCT in the SFI group was greater [WMD = −3.08, 95% CI: (−5.05, −1.12), *p* = 0.002]. The I^2^ value was 96%, and a random-effect model was utilized. The calculated WMD illustrated that SFI could reduce the PCT level of elderly severe pneumonia patients. Based on further analysis, two RCTs (Qu and Shao, 2018; Kong et al., 2022) were probably the sources of heterogeneity because the heterogeneity dropped from 96% to 0% after these studies were removed (I^2^ = 0).

##### 3.4.2.2 C-Reactive Protein

Figure 5B illustrated the effect of SFI on CRP (Lv et al., 2017; Wang, 2020; Kong et al., 2022). The pooled analysis, including 240 patients with a randomized model, found no significant difference on the CRP level between the SFI group and the control group with SMD −1.26 and 95% CI (−2.80, 0.28). The I^2^ value was 96%, and a random-effect model was used. According to sensitivity analysis conducted based on the risk of bias, the heterogeneity was probably brought by Wang’s study (Wang, 2020) with high selection bias because the I^2^ value was low to 24% when this outlier study was excluded. The *p*-value of the test for the overall effect was 0.11, and the current data revealed that SFI might not reduce the CRP level of elderly patients. Nonetheless, SFI may reduce the CRP level of the elderly patients based on the result of the subgroup of quasi RCTs [SMD = −3.12, 95% CI: (−3.79, −2.45), *p* < 0.00001].

##### 3.4.2.3 White Blood Cell Count

Figure 5C illustrated the effect of SFI on WBC pooling of two RCTs (Lv et al., 2017; Kong et al., 2022). The pooled analysis, including 162 participants, found a significant difference in the WBC count level between the SFI group and the control group with WMD −1.48 and 95% CI (−2.24, −0.72). A fixed-effect model was utilized since no heterogeneity was identified (I^2^ = 0). The *p*-value of the test for the overall effect was 0.0001, and the current data revealed that SFI might reduce the WBC of elderly patients with severe pneumonia.

#### 3.4.3 Cardiovascular System

##### 3.4.3.1 BNP Level

**FIGURE 6:**
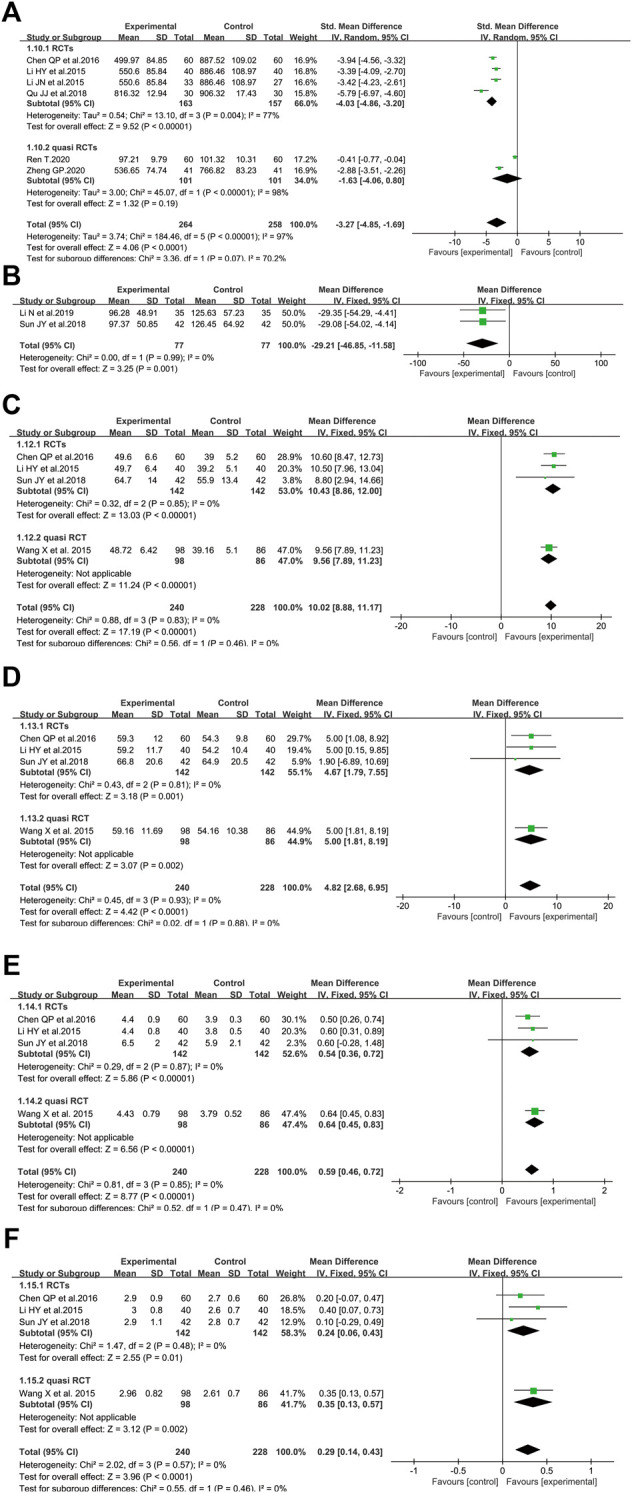
Meta-analysis results of SFI on the cardiovascular system based on: **(A)** BNP; **(B)** creatine Kinase; **(C)** LVEF; **(D)** stroke volume; **(E)** cardiac output; **(F)** cardiac index.

The BNP level was analyzed between SFI and non-SFI arms in six controlled trials involving 522 patients (Li et al., 2015a; Li et al., 2015b; Chen et al., 2016; Qu and Shao, 2018; Ren, 2020; Zheng, 2020) (Figure 6A). There were two studies (Li et al., 2015a; Chen et al., 2016) using μg/L as the measurement unit of BNP, while the measurement unit of three studies (Li et al., 2015b; Qu and Shao, 2018; Zheng, 2020) and one study (Ren, 2020) was ng/ml and pg/ml, respectively. Thus, the data utilized SMD to reduce the difference in measurement units. After standardization, the results demonstrated that the BNP of elderly severe pneumonia participants treated by SFI plus standard control was significantly decreased compared to those in the control group (SMD = −3.27, 95% CI = −4.85 to −1.69, *p* < 0.0001). A random-effect model was adopted to analyze SMD since the BNP level (*p* < 0.00001, I^2^ = 97%) was heterogeneous among the pooled studies. Similar results were identified in the subgroup of RCTs. Based on sensitivity analysis, two studies may have caused the heterogeneity (Qu and Shao, 2018; Ren, 2020) because the I^2^ value was less than 50% when these studies were excluded.

##### 3.4.3.2 Creatine Kinase Level

In terms of CK level, two RCTs involving 154 older participants with severe pneumonia reported SFI as an adjuvant vs. standard control alone (Sun et al., 2018; Li et al., 2019) (Figure 6B). The results revealed that the CK level was markedly decreased compared with the standard control alone (WMD = −29.21, 95% CI = −46.85 to −11.58, *p* = 0.001). Fixed-effect models were applied to calculate WMD since no heterogeneity was identified (*p* < 0.99, I^2^ = 0%).

##### 3.4.3.3 Left Ventricular Ejection Fraction

Figure 6C illustrated the effect of SFI on LVEF (Li et al., 2015a; Wang et al., 2015; Chen et al., 2016; Sun et al., 2018) with 240 in the SFI arm and 228 patients in the control arm. The LVEF of the SFI arm was improved (WMD = 10.02, 95% CI = 8.88–11.17, *p* < 0.00001) compared with that of the control arm. Data heterogeneity was not found and a fixed-effect model was conducted because the I^2^ value was 0%. The data demonstrated that SFI could improve the LVEF of elderly severe pneumonia patients.

##### 3.4.3.4 Stroke Volume

Four trials involving 468 patients with severe pneumonia evaluated the effect of SFI plus standard control versus standard control alone in boosting SV (Li et al., 2015a; Wang et al., 2015; Chen et al., 2016; Sun et al., 2018). The fixed-effect model was performed because of no heterogeneity identified among included studies (I^2^ = 0%, *p* = 0.93). The pooled results demonstrated that SFI as an adjuvant might improve SV compared with standard control alone for elderly severe pneumonia (WMD = 4.82, 95% CI: 2.68 to 6.95, *p* < 0.0001) (Figure 6D).

##### 3.4.3.5 Cardiac Output

Four eligible trials with 468 volunteers evaluated the CO level between the SFI arm and control arm (Li et al., 2015a; Wang et al., 2015; Chen et al., 2016; Sun et al., 2018). Given that no heterogeneity among these studies was identified (I^2^ = 0%, *p* = 0.85), a meta-analysis was conducted utilizing a fixed-effect model. SFI as an adjuvant could significantly increase the CO level compared to standard control alone (WMD = 0.59, 95% CI: 0.46–0.72, *p* < 0.00001, Figure 6E).

##### 3.4.3.6 Cardiac Index

Regarding CI, four trials with 468 subjects investigated the effect of SFI plus standard control on older patients with severe pneumonia (Li et al., 2015a; Wang et al., 2015; Chen et al., 2016; Sun et al., 2018). A fixed-effect model was adopted to conduct the meta-analysis since no heterogeneity was identified (I^2^ = 0%, *p* = 0.57). SFI plus standard control achieved a greater improvement for elderly severe pneumonia patients (Figure 6F) when compared with standard control alone (WMD = 0.29, 95% CI: 0.14–0.43, *p* < 0.0001).

#### 3.4.4 Markers of Endothelial Perturbation

##### 3.4.4.1 Soluble Endothelial Selectin Level

**FIGURE 7:**
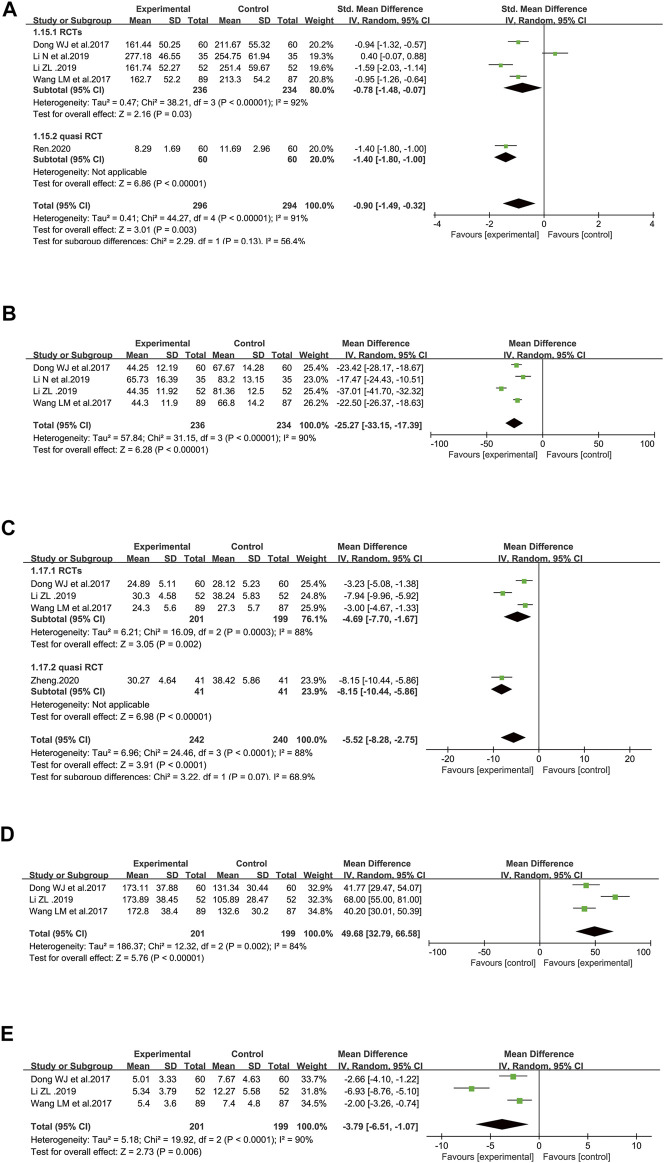
Meta-analysis results of markers of endothelial perturbation and coagulation dysfunction based on: **(A)** sE-selectin; **(B)** von Willebrand factor; **(C)** APTT; **(D)** platelet counts; **(E)** D-Dimer.

There were five studies that reported sE-selectin, totaling 590 patients (296 cases in the SFI arm and 294 cases in the control arm) (Dong et al., 2017; Wang et al., 2017; Li, 2019; Li et al., 2019; Ren, 2020). The level of sE-selectin was lower in the SFI groups [SMD = −0.90, 95% CI (−1.49 to −0.32), *p* = 0.003] when compared with that of standard control alone. A random-effect model was used since substantial heterogeneity (I^2^ = 91%) was observed among the results. Similar results were shown in the subgroups analysis based on the study design (Figure 7A). According to further analysis, the heterogeneity may be caused by two studies (Li, 2019; Li et al., 2019) because the I^2^ value was less than 50% when these studies were removed.

##### 3.4.4.2 Von Willebrand Factor Level

Figure 7B illustrated the efficacy of SFI on the vWF level with 470 patients included (236 in the SFI arm and 234 participants in the control group) in four RCTs (Dong et al., 2017; Wang et al., 2017; Li, 2019; Li et al., 2019) using a randomized model because of heterogeneity (I^2^ = 90%) that was probably induced by one study (Li, 2019), considering that the I^2^ value was only 2% when this study was removed. The decrease in the vWF level was greater in the SFI arm than in the control arm [WMD = −25.27, 95% CI: (−33.15, −17.39), *p* < 0.00001]. The data indicated that SFI as an adjuvant might achieve a better effect on reducing the vWF level of elderly patients with severe pneumonia.

#### 3.4.5 Coagulation Dysfunction

##### 3.4.5.1 Activated Partial Thromboplastin Time

Figure 7C illustrated the effect of SFI on APTT, totaling 482 patients (242 participants in the SFI arm and 240 participants in the control arm) using a randomized model in four included studies (Dong et al., 2017; Wang et al., 2017; Li, 2019; Zheng, 2020). The reduction in APTT was greater in the SFI arm than in the control arm [WMD = −5.52, 95% CI: −8.28, −2.75), *p* < 0.0001]. A randomized model was utilized because of heterogeneity (I^2^ = 88%), which may be brought by two studies (Li, 2019; Zheng, 2020), since the I^2^ value was low to zero after these studies were removed. The pooled results demonstrated that SFI as an adjuvant could achieve a better effect on decreasing APTT in elderly patients with severe pneumonia. Similar results were illustrated in the subgroup analysis based on the study design.

##### 3.4.5.2 Platelet Counts

Figure 7D illustrated the effect of SFI on platelet counts with totally 400 patients (201 participants in the SFI arm and 199 participants in the control arm) based on three RCTs (Dong et al., 2017; Wang et al., 2017; Li, 2019). SFI plus standard control achieved a greater improvement in platelet counts than the standard control (WMD = 49.68, 95% CI: 32.79–66.58, *p* < 0.00001). A randomized model was utilized since the I^2^ value (84%) indicated significant heterogeneity, which may be brought by Li’s study (Li, 2019) since the I^2^ value was low to zero after this study was removed.

##### 3.4.5.3 D-Dimer

Figure 7E illustrated the effect of SFI on D-Dimer with 400 patients included (201 participants in the SFI arm and 199 in the control arm) based on three RCTs (Dong et al., 2017; Wang et al., 2017; Li, 2019). A randomized model was adopted due to significant heterogeneity (I^2^ = 90%, *p* < 0.0001). Based on further analysis, the heterogeneity may be caused by Li’s study (Li, 2019) since the I^2^ value was low to zero after this study was removed. The chart illustrated that SFI as an adjuvant could decrease D-Dimer better for elderly participants with severe pneumonia [WMD = −3.79, 95% CI: −6.51, −1.07), *p* < 0.0001].

### 3.5 Sensitivity Analysis

Sensitivity analysis was implemented for the outcome with heterogeneity, and relevant results have been demonstrated in the individual section above. In addition, we tested the robustness of all results. As a result, the conclusions for most outcomes, including total effective rate, BNP, APACHE II score, sE-selectin, Vwf, APTT, platelet counts, PaO_2_, lactic acid accumulation, WBC, CRP, and PCT, were stable because the combined RR, WMD, or SMD of overall risk estimates were consistent, without reversion after deleting a single study each time. Therefore, the sensitivity analyses based on those outcomes gave results very close to the main results, verifying that SFI may be beneficial for improving total effective rate, BNP, APACHE II score, sE-selectin, Vwf, APTT, platelet counts, PaO_2_, lactic acid accumulation, WBC count, CRP, and PCT. Nonetheless, the sensitivity analysis of D-Dimer demonstrated that those results were not robust enough and needed to be further verified since the individual study significantly affected pooled results.

### 3.6 Publication Bias

Given that none of the above meta-analyses involved more than ten studies and the inclusive studies were inadequate to generate funnel plots, publications bias was not assessed to screen publication bias.

### 3.7 The Certainty of Evidence

According to the GRADE approach, the certainty of findings of the effect of SFI on LVEF, SV, CO, and CI based on RCTs was high. The certainty of findings relevant to the effective rate, APACHE-II, mortality rate, safety profiles, lactic acid accumulation, CRP, WBC, CK, and platelet counts based on RCTs was moderate. The certainty of evidence about the effect of SFI on APACHE II, mortality rate, safety profiles, PaO_2_, lactic acid accumulation, PCT, CRP, LVEF, SV, CO, CI, sE-selectin, and APTT based on quasi RCTs and PaO_2_, PCT, BNP, sE-selectin, Vwf, APTT, and D-Dimer based on RCTs was low (Supplementary Table S3).

## 4 Discussion

### 4.1 Overview

Managing severe pneumonia in older adults is critical for the treatment development of COVID-19 in the elderly as the elderly are disproportionately affected by severe pneumonia. Given that there are currently no evidence-based recommendations for elderly severe COVID-19 and studies on the optimal treatments for older patients with severe or fatal pneumonia are lacking, the present study is necessary. It may give some hints on these issues. Here, we briefly reviewed the SFI aimed at the older population, focusing on the evidence of safety and efficacy, and various laboratory biomarkers associated with COVID-19 disease progression. In China, SFI has been applied routinely in treating critically ill patients with fatal diseases such as shock, coronary heart disease, congestive heart failure, and trauma, with a usage history of more than 30 years for end-stage diseases (Zhang et al., 2012; Huang and Cao, 2014). SFI, which mainly consists of ginsenoside and aconite total alkaloids, has been registered in China’s State Food and Drug Administration and has undergone rigorous scientific and clinical scrutiny to establish efficacy and safety. In the present study, we have provided a complete chain of evidence to determine the effectiveness and safety of SFI on elderly patients with severe pneumonia in related clinical and experimental profiles. First, SFI might improve the total effective rate and the APACHE II score and reduce safety concerns and mortality rate. Second, improving oxygenation played a vital role in decreasing mortality, and SFI could improve PaO_2_ and decrease lactic acid accumulation levels based on the present study. The primary clinical presentation of severe COVID-19 was an acute respiratory failure with extreme hypoxemia, which ultimately required mechanical ventilation (Berlin et al., 2020). A recent study revealed that elderly patients with severe pneumonia including COVID-19 were at risk for acute respiratory distress syndrome (Grasselli et al., 2020). Among elderly patients with COVID-19, severe hypoxia and lactic acid accumulation resulting from respiratory failure may increase the risk of death, contributing to poor outcomes (Peng et al., 2020). The therapeutic effect of SFI on reducing mortality and improving clinical outcomes including total effective rate and the APACHE II score may be achieved through improving gas exchange during severe pneumonia, which deserves to be investigated further on COVID-19. Third, PCT played a principal role in severe cases of COVID-19 (Bhargava et al., 2020; Hu et al., 2020), especially in COVID ICU patients (Grasselli et al., 2020). SFI might decrease PCT levels based on our studies including patients with severe pneumonia, providing indirect evidence for further trials on COVID-19. Fourth, the effect of SFI was verified from the cardiovascular system, endothelial function, and coagulation function. While elderly patients with severe pneumonia primarily suffered from lung injury with interstitial pneumonitis and severe acute respiratory distress syndrome, the cardiovascular system was also affected, especially in elderly COVID-19 patients (Guzik et al., 2020). Mechanistically, after proteolytic cleavage of its S protein by a serine protease, SARS-CoV-2 binds to the transmembrane angiotensin-converting enzyme 2 (ACE2) to enter type 2 pneumocytes, macrophages, perivascular pericytes, and cardiomyocytes, contributing to microvascular and endothelial dysfunction, plaque instability, myocardial dysfunction and damage, and myocardial infarction (Guzik et al., 2020). There has been increasing evidence that severe pneumonia, including COVID-19, may lead to severe cardiac involvement with cardiac dysfunction and elevation of myocardial injury markers including BNP and CK levels (Zheng et al., 2020), which were demonstrated to be related to poor prognosis (Rath et al., 2020). CO and cardiac SV were considered determinants of epidemic fatality (Stevens, 1976), and low SV and CO conveyed the information about the patients’ bad hemodynamic status and cardiovascular system function (Kamoi et al., 2014). Previous studies have also illustrated higher D-Dimer levels in critically ill or fatal cases, especially in patients with SARS-COV-2 infection (Zhou et al., 2020). In addition, markers of endothelial perturbation, namely, sE-selectin and vWF have been reported to be proportional to the severity of pneumonia (Cugno et al., 2021). COVID-19 could cause a hypercoagulable state and coagulation dysfunction, which were more likely to occur in severe and critically ill patients. The levels of markers of coagulation activation played a significant role in predicting the severity and prognosis of COVID-19, and APTT might be applied as indicators in predicting the mortality of COVID-19 (Long et al., 2020). SFI may alleviate cardiac injury by decreasing the BNP level, CK level, sE-selectin, vWF, APTT, and D-Dimer level and improving LVEF, SV, CO, and CI during severe pneumonia with fewer safety concerns according to the present pooled results. Although ideal treatments for severe COVID-19 have not been available, the end-stage of fatal COVID-19 is similar to the end-stage of severe pneumonia since the imminent tasks for rescuing the patients clinically are improving gas exchange, strengthening heart function, and reducing inflammation, endothelial perturbation, and coagulation dysfunction. Whether SFI might be a potentially promising choice in rescuing critical or fatal COVID-19 patients deserves further clinical trials.

### 4.2 Mechanism of Shenfu Injection Based on “WE” Medicine

The widespread use of SFI is the application of “WE” medicine. Shenfu decoction, the origin of SFI, was first recorded in “The Yan Family’s Recipe for Health” (Yanshi Jisheng Fang) in the Southern Song Dynasty (1253 AD) and was suggested in emergency treatments as the classic Chinese formula for centuries. Pneumonia in the elderly was a severe problem with clinical presentations that differed from those in younger patients. Based on TCM, the elderly people with insufficient healthy-qi and visceral deficiency were vulnerable to depletion of yin and yang (Zhang et al., 2021), which was the pathological basis of severe pneumonia and played a vital role in the development and prognosis of COVID-19, tending to result in sudden death. Since the ability of elderly patients to resist the pathogenetic factors declined, invigorating yang-qi promptly for elderly severe pneumonia patients with loss of yang was clearly warranted. With the development of integrated traditional and western medicine (Qiu et al., 2020), it was believed that SFI might be more suitable for elderly severe pneumonia patients than decoction since older patients complained of more difficulty swallowing than younger patients and were more susceptible to coma with the inability to take oral medications (J., 2000). SFI was the empirical treatment of patients with the sudden exhaustion of yang-qi in clinical practice. The elderly severe pneumonia patients might present with few respiratory symptoms and signs. Instead, they could be manifest as delirium, worsening chronic confusion, and even coma (Yoshikava and Marrie, 2000), which could be rescued by SFI, especially those with sweating, cold limbs, purplish-gray ash tongue, and pulse that is floating, large, and weak. Cardiac SV could determine the epidemic fatality (Stevens, 1976), and low SV was prevalent in critical care (Kamoi et al., 2014). The SV value in the SFI arm may be dramatically higher than that of the control arm since the severe pneumonia patients’ response to SFI therapy was positive based on our results, which might also explain why SFI improved the hemodynamic profiles.

Age-related decline such as immunosenescence and inflammaging was able to heighten the vulnerability of elderly patients to severe COVID-19 (Chen et al., 2021). Understanding the theory from modern medicine, there are antiaging active ingredients in SFI that have beneficial effects on anti-oxidation, restoration of function, and reducing the deleterious contribution of senescent cells in aged diseases (Li et al., 2016). Ginsenoside, the main component of SFI, could inhibit the activation of the Wnt/β-catenin signaling pathway and decrease the senescence of neural stem cells (NSCs) (Xiang et al., 2019).

SFI, designed for the specific clinical presentation in severe and fatal cases, has been used widely in treating critically ill patients with respiratory failure and elderly severe pneumonia. Integrating the concepts of TCM with modern medicine could be an effective approach toward the overall management of elderly severe COVID-19 in the absence of specific drugs to treat this pandemic (Huang et al., 2020; Qiu et al., 2020). The protective effects of SFI against severe pneumonia have been researched by clinical studies for multiple indications and have been extensively studied through different experimental models. Indeed, SFI involved multiple targets, and the potential mechanism might be relevant to improving antioxidant capacity and energy metabolism and delaying cell senescence (Xiang et al., 2019). SFI infusion or the main component of SFI could enhance the activity of superoxide dismutase, Ca^2+^-ATPase, Na^+^-K^+^-ATPase, and reduce the malondialdehyde content of lung tissue (Zhang et al., 2012). Recent studies have also reported that SFI may inhibit the activity of IL-6 and TNF-**α** and alleviate acute lung injury *via* reducing the expression of NF-kB (Wang et al., 2008). Furthermore, SFI can protect lung tissue, inhibit heart failure, reduce the histological damage, and play an anti-inflammatory effect by altering the balance of microRNAs involved in activating and inhibiting apoptosis, regulating the production of cytokines, and inhibiting the expression of NF-kB (Huang and Cao, 2014; Yan et al., 2018). Given that the lung was responsible for pulmonary gas exchange, these pulmonary protective effects of SFI may simultaneously contribute to the recovery of vital organs including the heart and brain, thereby improving severe and fatal cases (Zhang et al., 2012). The molecular mechanisms by which SFI treats the elderly with severe COVID-19 remain elusive and merit exploration in the future.

### 4.3 Safety

A critical issue regarding the empirical treatment of SFI is that physicians ought to pay careful attention to safety and drug interactions. The most frequent safety concerns reported in included studies were adverse cardiovascular events (Ren, 2020). Although negative symptoms of the SFI group were dramatically less than those of the control group according to our results, the safety of the intervention should not be neglected. Based on the eighteen incompatibilities and nineteen medicaments of mutual restraint in TCM, SFI should not be used with *Pinellia ternata* (Thunb.) Makino, *Trichosanthes rosthornii* Harms, *Trichosanthes kirilowii* Maxim, *Fritillaria usuriensis* Maxim, *Fritillaria cirrhosa* D. Don, *Fritillaria pallidiflora* Schrenk, *Fritillaria thunbergii* Miq., *Fritillaria monantha* Migo, *Fritillaria unibracteata* P. K. Hsiao & K. C. Hsia, *Ampelopsis japonica* (Thunb.) Makino, or *Bletilla striata* (Thunb.) Rchb. F. Nevertheless, toxicological data on the drug interactions are inadequate, and the mechanisms are still largely unknown, which may lead to harming patients (Heinrich et al., 2020). Therefore, high-quality findings on drug interactions are warranted for the future benefit of severe pneumonia patients and for the development of SFI.

### 4.4 Limitations and Implications for Further Research

Considering that older patients generally have dysfunction of multiple organs due to the aging process and comorbidity, physicians are supposed to pay attention to drug dosages and optimal total duration in this group. Apart from giving SFI based on patient illness states and physical conditions by convention, 7–14 days was the usual duration of therapy based on the present systematic review. However, this review did not identify the best dosages and optimal total duration of SFI for elderly patients with severe COVID-19, which has not been explored in available prospective studies. Clinical trials are supposed to be conducted to determine if a longer duration is necessary or beneficial for elderly patients. In addition, severe pneumonia in elderly patients is often combined with multiple diseases. Subgroup analysis may be conducted in the future to assess the therapeutic effect of SFI on a single disease. Moreover, the impact of risk of bias on the certainty of evidence should not be underestimated, which was considered and interpreted in the table generated by the GRADEprofiler, leading to downgrading of the evidence for corresponding outcomes. Additionally, the heterogeneity of a few outcomes was an unavoidable issue, and definitive conclusions about heterogeneity were hard to be made. We have investigated the main sources of heterogeneity, which may be study design, based on sensitivity analysis. For instance, the heterogeneity of PCT and BNP brought by Qu’s RCT (Qu and Shao, 2018) was probably because the age range was different from that in other included studies, and the heterogeneity of PCT brought by Kong’s study (Kong et al., 2022) was probably due to the different conventional pharmacotherapy, and the heterogeneity of CRP may come from the risk of bias according to the opinions from clinical experts in our team (WL and FW). However, further trials specifically assessing those points were needed to confirm whether those factors contributed to the differences in the meta-analyses results. In addition, international guidelines regarding the use of SFI in elderly patients with severe pneumonia from different regions and ethnicities are not available. The cooperation of various countries is required to investigate the effect of SFI since the metabolism of SFI might be different in various races. Furthermore, the results and conclusion about a few outcomes with limited sample size might be changed in the future since the understanding of SFI treating severe pneumonia has been evolving continuously. Therefore, we intend to explore these unstable outcomes in our future research. According to the National Health Commission of the People’s Republic of China (PRC) guidance (The Chinese National Health Commission, 2020), SFI plus standard control in severe pneumonia was recommended based on clinical practice but without an evidence-based reference for the rational use of SFI. The present study may fill the gap in evidence-based medicine. Nonetheless, much remains to be learned about the treatment for elderly severe COVID-19, including the minimal clinically important difference (MCID), which is unknown for most outcomes, and whether the magnitude of these effects represents MCID has not been investigated. MCID, an approach to assess the clinical significance (Bennett, 2016), has not been widely used in severe pneumonia research, affecting the results’ external validity, including the markers of endothelial perturbation and clinical relevance. We hope that more data about recommended levels for the MCID based on changes during severe pneumonia, especially COVID-19, will be available to verify the relevant clinical benefits.

## 5 Conclusion

SFI combined with standard control might reduce mortality and safety concerns and improve the total effective rate, APACHE II score, oxygenation, gas exchange, and predictors associated with COVID-19 disease progression including BNP level, the CK level, sE-selectin, vWF level, APTT, platelet counts, D-Dimer, WBC, and PCT for elderly patients with severe pneumonia based on evidence with moderate-to-low certainty. SFI as an adjuvant may improve LVEF, SV, CO, and CI for this group as well, based on evidence with high certainty. SFI might have protective effects against elderly severe pneumonia in many aspects, including hematological, inflammatory, immunological, and biochemical markers. To sum up, SFI may have curative effects in treating the elderly with severe pneumonia, which is indirect evidence for further trials on COVID-19. However, large, multi-center RCTs are clearly warranted to confirm these findings further.

## Data Availability

The original contributions presented in the study are included in the article/Supplementary Material; further inquiries can be directed to the corresponding authors.
