# Supplementary material for: Efficacy and Safety of Shenfu Injection for Severe Pneumonia in the Elderly: A Systematic Review and Meta-Analysis Based on Western and Eastern Medicine
Source: Front Pharmacol. 2022 Aug 25;13:779942. doi: 10.3389/fphar.2022.779942 (PMC9454296; doi:10.3389/fphar.2022.779942)
Supplement: Supplementary file 2 [file Table2.docx]

**Search strategies**

| Literature databases | Search items | Items found |
| --- | --- | --- |
| (1) PubMed | (("coronavirus disease 2019" OR "COVID-19" OR "severe acute respiratory syndrome coronavirus 2" OR "SARS-CoV-2" OR "coronavirus" OR "novel coronavirus" OR "nCoV" OR "2019-nCoV" OR "pneumonia" OR "Severe Acute Respiratory Syndrome"[MeSH Terms]) OR ("coronavirus disease 2019" OR "COVID-19" OR "severe acute respiratory syndrome coronavirus 2" OR "SARS-CoV-2" OR "coronavirus" OR "novel coronavirus" OR "nCoV" OR "2019-nCoV" OR "severe pneumonia" OR "Severe Acute Respiratory Syndrome")) AND ("Shenfu Injection" OR "SFI" OR "Shenfu" OR "Shenfu decoction" OR "SF injection" OR "SF" OR "ginsenoside" OR "aconite total alkaloids" OR " Panax ginseng C. A. Meyer" OR "Radix Aconitum Carmichaeli") | 432 |
| (2)Science Direct | #1  Title, abstract, keywords: "coronavirus disease 2019" OR "COVID-19" OR "coronavirus" OR "novel coronavirus" OR "pneumonia" OR "severe pneumonia" OR "serious pneumonia"  #2  "Shenfu Injection" OR "SFI" OR "Shenfu" OR "Shenfu decoction"OR "SF injection" OR "SF" OR "ginsenoside"OR "aconite total alkaloids"  #1 AND #2 | 1079 |
| (3)Web of science | ALL=("coronavirus disease 2019" OR "COVID-19" OR "coronavirus" OR "novel coronavirus" OR "pneumonia" OR "severe pneumonia" OR "serious pneumonia") AND ALL=("Shenfu Injection" OR "SFI" OR "Shenfu")  Timespan: All years. Indexes: SCI-EXPANDED. | 45 |
| (4)Google Scholar | ("coronavirus disease 2019" OR "COVID-19" OR "coronavirus" OR "novel coronavirus" OR "severe pneumonia" OR "serious pneumonia") AND ("Shenfu Injection" OR "SFI" OR "Shenfu") | 2,750 |
| (5)Embase | #1 'severe acute respiratory syndrome coronavirus 2'/exp  #2 'coronavirus disease 2019' OR 'covid-19' OR 'severe acute respiratory syndrome coronavirus 2' OR 'sars-cov-2' OR 'coronavirus' OR 'novel coronavirus' OR 'ncov' OR '2019-ncov' OR 'severe pneumonia' OR 'severe acute respiratory syndrome':ab  #3 #1 OR #2  #4 'shenfu injection' OR 'sfi' OR 'shenfu' OR 'shenfu decoction' OR 'sf injection' OR 'sf' OR 'ginsenoside' OR 'aconite total alkaloids' OR 'panax ginseng c. a. meyer' OR 'radix aconitum carmichaeli' OR 'ginsenosides'  #5 #3 AND #4 | 327 |
| (6)China National Knowledge Infrastructure Database | #1  (主题="新型冠状病毒肺炎") OR (主题="新冠肺炎") OR (主题="COVID-19") OR (主题="2019 冠状病毒病") OR (主题="冠状病毒肺炎") OR (主题="肺炎")  #2  (篇关摘="参附注射液") OR (篇关摘="参附汤") OR (篇关摘="参附")  #1 AND #2 | 92 |
| (7)Wan Fang database Search strategy | 主题:( "重症新型冠状病毒肺炎" or "新冠肺炎重症" or "重症COVID-19" or "2019 冠状病毒病重症" or "重症冠状病毒肺炎" or "重症肺炎") and 全部:( "参附注射液" or "参附汤" or "参附") | 34 |
| (8)Chongqing VIP Chinese Science and Technology Periodical Database (VIP) | #1  任意字段=参附注射液 OR 任意字段=参附汤) OR 任意字段=参附  #2  任意字段=重症新型冠状病毒肺炎 OR 任意字段=新冠肺炎重症 OR 任意字段=2019 冠状病毒病重症 OR 任意字段=重症冠状病毒肺炎 OR 任意字段=重症肺炎  #1 AND #2 | 38 |
| (9) SinoMed | ( "参附注射液"[关键词:智能] OR "参附汤"[关键词:智能] OR "参附"[关键词:智能]) AND( "新型冠状病毒肺炎"[全部字段:智能] OR "新冠肺炎"[全部字段:智能] OR "COVID-19"[全部字段:智能] OR "2019 冠状病毒病"[全部字段:智能] OR "冠状病毒肺炎"[全部字段:智能] OR "肺炎"[全部字段:智能] | 27 |
| Overall |  | 4,824 |
