# Supplementary material for: Efficacy and Safety of Shenfu Injection for Severe Pneumonia in the Elderly: A Systematic Review and Meta-Analysis Based on Western and Eastern Medicine
Source: Front Pharmacol. 2022 Aug 25;13:779942. doi: 10.3389/fphar.2022.779942 (PMC9454296; doi:10.3389/fphar.2022.779942)
Supplement: Supplementary file 3 [file Table3.docx]

**TABLE 3.** The certainty of evidence

TABLE 3.1. The certainty of evidence for primary outcomes

| **Shenfu injection plus standard control compared to standard control for elderly severe pneumonia** | | | | | | |
| --- | --- | --- | --- | --- | --- | --- |
| **Patient or population:** Patients with elderly severe pneumonia **Settings:** Randomized controlled trials (RCT) or quasi RCTs **Intervention:** Shenfu injection plus standard control **Comparison:** Standard control | | | | | | |
| **Outcomes** | **Illustrative comparative risks* (95% CI)** | | **Relative effect (95% CI)** | **No of Participants (studies)** | **Quality of the evidence (GRADE)** | **Comments**^5^ |
|  | Assumed risk | Corresponding risk |  |  |  |  |
|  | **standard control** | **Shenfu injection plus standard control** |  |  |  |  |
| **Effective rate - RCTs** | **Study population** | | **RR 1.19**  (1.06 to 1.33) | 307 (5 studies) | ⊕⊕⊕⊝ **moderate**^1^ |  |
|  | **694 per 1000** | **826 per 1000** (736 to 923) |  |  |  |  |
|  | **Moderate** | |  |  |  |  |
|  | **750 per 1000** | **893 per 1000** (795 to 998) |  |  |  |  |
| **Effective rate - quasi RCTs** | **Study population** | | **RR 1.32**  (1.15 to 1.51) | 382 (3 studies) | ⊕⊝⊝⊝ **very low**^2^ |  |
|  | **551 per 1000** | **728 per 1000** (634 to 833) |  |  |  |  |
|  | **Moderate** | |  |  |  |  |
|  | **769 per 1000** | **1000 per 1000** (884 to 1000) |  |  |  |  |
| **APACHE II - RCTs** |  | The mean apache ii - rcts in the intervention groups was **2.95 lower** (3.35 to 2.56 lower) |  | 809 (8 studies) | ⊕⊕⊕⊝ **moderate**^1^ |  |
| **APACHE II - quasi RCT** |  | The mean apache ii - quasi rct in the intervention groups was **6.43 lower** (7.61 to 5.25 lower) |  | 82 (1 study) | ⊕⊕⊝⊝ **low** |  |
| **Mortality Rate - RCTs** | **Study population** | | **RR 0.55**  (0.35 to 0.84) | 245 (4 studies) | ⊕⊕⊕⊝ **moderate**^1^ |  |
|  | **351 per 1000** | **193 per 1000** (123 to 295) |  |  |  |  |
|  | **Moderate** | |  |  |  |  |
|  | **366 per 1000** | **201 per 1000** (128 to 307) |  |  |  |  |
| **Mortality Rate - quasi RCT** | **Study population** | | **RR 0.48**  (0.28 to 0.83) | 184 (1 study) | ⊕⊕⊝⊝ **low** |  |
|  | **337 per 1000** | **162 per 1000** (94 to 280) |  |  |  |  |
|  | **Moderate** | |  |  |  |  |
|  | **337 per 1000** | **162 per 1000** (94 to 280) |  |  |  |  |
| **Adverse events - RCT** | **Study population** | | **RR 0.3**  (0.17 to 0.53) | 120 (1 study) | ⊕⊕⊕⊝ **moderate**^1^ |  |
|  | **617 per 1000** | **185 per 1000** (105 to 327) |  |  |  |  |
|  | **Moderate** | |  |  |  |  |
|  | **617 per 1000** | **185 per 1000** (105 to 327) |  |  |  |  |
| **Adverse events - quasi RCT** | **Study population** | | **RR 0.25**  (0.03 to 1.98) | 30 (1 study) | ⊕⊕⊝⊝ **low**^3,4^ |  |
|  | **267 per 1000** | **67 per 1000** (8 to 528) |  |  |  |  |
|  | **Moderate** | |  |  |  |  |
|  | **267 per 1000** | **67 per 1000** (8 to 529) |  |  |  |  |
| *The basis for the **assumed risk** (e.g., the median control group risk across studies) is provided in footnotes. The **corresponding risk** (and its 95% confidence interval) is based on the assumed risk in the comparison group and the **relative effect** of the intervention (and its 95% CI).  **CI:** Confidence interval; **RR:** Risk ratio; | | | | | | |
| GRADE Working Group grades of evidence **High quality:** Further research is very unlikely to change our confidence in the estimate of effect.  **Moderate quality:** Further research is likely to have an important impact on our confidence in the estimate of effect and may change the estimate. **Low quality:** Further research is very likely to have an important impact on our confidence in the estimate of effect and is likely to change the estimate. **Very low quality:** We are very uncertain about the estimate. | | | | | | |
| ^1^ Random sequence generation and the blinding methods were unclear, and we decided to downgrade the quality of evidence as risk of bias.  ^2^ There was serious heterogeneity among the studies included. Overall, we decided to downgrade by one level.  ^3^ Results are imprecise since studies include relatively few patients and few events and thus have wide confidence intervals around the estimate of the effect.  ^4^ RR <0.5 based on consistent evidence from at least 2 studies  ^5^ Explanations about why three domains of ROB assessments were not sufficient for downgrading the certainty of evidence:  Incomplete accounting of patients and outcome events, Selective outcome reporting, and Other limitations:  All trials studying SFI for severe pneumonia were conducted in hospital and included inpatients during the treatments, and there was no loss to follow-up and failure to adhere to the intention to treat principle when indicated with complete medical records. Besides, the treatment duration of SFI was based on standard drug instructions, restricted to requirements of medical ethics, and supervised by hospitals, thus, stopping early for benefit observed in randomized trials, in particular in the absence of adequate stopping rules use of unvalidated patient-reported outcomes, and reporting of some outcomes and not others on the basis of the results were nearly impossible. Therefore, the quality of the evidence for theses domains may not be downgraded. | | | | | | |

TABLE 3.2**.** The certainty of evidence for secondary outcomes

| **Shenfu injection plus standard control compared to standard control for elderly severe pneumonia** | | | | | | |
| --- | --- | --- | --- | --- | --- | --- |
| **Patient or population:** Patients with elderly severe pneumonia **Settings:** Randomized controlled trials (RCT) or quasi RCTs **Intervention:** Shenfu injection plus standard control **Comparison:** Standard control | | | | | | |
| **Outcomes** | **Illustrative comparative risks* (95% CI)** | | **Relative effect (95% CI)** | **No of Participants (studies)** | **Quality of the evidence (GRADE)** | **Comments**^3^ |
|  | Assumed risk | Corresponding risk |  |  |  |  |
|  | **standard control** | **Shenfu injection plus standard control** |  |  |  |  |
| **Partial pressure of arterial oxygen (PaO2) - RCTs** |  | The mean partial pressure of arterial oxygen (pao2) - rcts in the intervention groups was **15.93 higher** (12.8 to 19.06 higher) |  | 231 (4 studies) | ⊕⊕⊝⊝ **low**^1,2^ |  |
| **Partial pressure of arterial oxygen (PaO2) - quasi RCT** |  | The mean partial pressure of arterial oxygen (pao2) - quasi rct in the intervention groups was **18.3 higher** (15.87 to 20.73 higher) |  | 90 (1 study) | ⊕⊕⊝⊝ **low** |  |
| **Lactic acid accumulation - RCTs** |  | The mean lactic acid accumulation - rcts in the intervention groups was **1.41 lower** (2.23 to 0.58 lower) |  | 79 (2 studies) | ⊕⊕⊕⊝ **moderate**^1^ |  |
| **Lactic acid accumulation - quasi RCT** |  | The mean lactic acid accumulation - quasi rct in the intervention groups was **1.44 lower** (2.06 to 0.82 lower) |  | 90 (1 study) | ⊕⊕⊝⊝ **low** |  |
| **Procalcitonin (PCT) - RCTs** |  | The mean procalcitonin (pct) - rcts in the intervention groups was **2.29 lower** (4.47 to 0.12 lower) |  | 193 (3 studies) | ⊕⊕⊝⊝ **low**^1,2^ |  |
| **Procalcitonin (PCT) - quasi RCTs** |  | The mean procalcitonin (pct) - quasi rcts in the intervention groups was **4.09 lower** (4.62 to 3.56 lower) |  | 160 (2 studies) | ⊕⊕⊝⊝ **low** |  |
| **C-reactive protein (CRP) - RCT** |  | The mean c-reactive protein (crp) - rct in the intervention groups was **0.35 standard deviations lower** (0.71 lower to 0.01 higher) |  | 162 (2 studies) | ⊕⊕⊕⊝ **moderate**^1^ | SMD -0.35 (-0.71 to 0.01) |
| **C-reactive protein (CRP) - quasi RCT** |  | The mean c-reactive protein (crp) - quasi rct in the intervention groups was **3.12 standard deviations lower** (3.79 to 2.45 lower) |  | 78 (1 study) | ⊕⊕⊝⊝ **low** | SMD -3.12 (-3.79 to -2.45) |
| **WBC- RCTs** |  | The mean wbc in the intervention groups was **1.48 lower** (2.24 to 0.72 lower) |  | 162 (2 studies) | ⊕⊕⊕⊝ **moderate**^1^ |  |
| **BNP - RCTs** |  | The mean bnp - rcts in the intervention groups was **4.03 standard deviations lower** (4.86 to 3.2 lower) |  | 320 (4 studies) | ⊕⊕⊝⊝ **low**^1,2^ | SMD -4.03 (-4.86 to -3.2) |
| **BNP - quasi RCTs** |  | The mean bnp - quasi rcts in the intervention groups was **1.63 standard deviations lower** (4.06 lower to 0.8 higher) |  | 202 (2 studies) | ⊕⊝⊝⊝ **very low**^2^ | SMD -1.63 (-4.06 to 0.8) |
| **CK- RCTs** |  | The mean ck in the intervention groups was **29.21 lower** (46.85 to 11.58 lower) |  | 154 (2 studies) | ⊕⊕⊕⊝ **moderate**^1^ |  |
| **Left ventricular ejection fraction (LVEF) - RCTs** |  | The mean left ventricular ejection fraction (lvef) - rcts in the intervention groups was **10.43 higher** (8.86 to 12 higher) |  | 284 (3 studies) | ⊕⊕⊕⊕ **high** |  |
| **Left ventricular ejection fraction (LVEF) - quasi RCT** |  | The mean left ventricular ejection fraction (lvef) - quasi rct in the intervention groups was **9.56 higher** (7.89 to 11.23 higher) |  | 184 (1 study) | ⊕⊕⊝⊝ **low** |  |
| **Stroke Volume (SV) - RCTs** |  | The mean stroke volume (sv) - rcts in the intervention groups was **4.67 higher** (1.79 to 7.55 higher) |  | 284 (3 studies) | ⊕⊕⊕⊕ **high** |  |
| **Stroke Volume (SV) - quasi RCT** |  | The mean stroke volume (sv) - quasi rct in the intervention groups was **5 higher** (1.81 to 8.19 higher) |  | 184 (1 study) | ⊕⊕⊝⊝ **low** |  |
| **Cardiac output (CO) - RCTs** |  | The mean cardiac output (co) - rcts in the intervention groups was **0.54 higher** (0.36 to 0.72 higher) |  | 284 (3 studies) | ⊕⊕⊕⊕ **high** |  |
| **Cardiac output (CO) - quasi RCT** |  | The mean cardiac output (co) - quasi rct in the intervention groups was **0.64 higher** (0.45 to 0.83 higher) |  | 184 (1 study) | ⊕⊕⊝⊝ **low** |  |
| **Cardiac output index (CI) - RCTs** |  | The mean cardiac output index (ci) - rcts in the intervention groups was **0.24 higher** (0.06 to 0.43 higher) |  | 284 (3 studies) | ⊕⊕⊕⊕ **high** |  |
| **Cardiac output index (CI) - quasi RCT** |  | The mean cardiac output index (ci) - quasi rct in the intervention groups was **0.35 higher** (0.13 to 0.57 higher) |  | 184 (1 study) | ⊕⊕⊝⊝ **low** |  |
| **sE-selectin - RCTs** |  | The mean se-selectin - rcts in the intervention groups was **0.95 standard deviations lower** (1.19 to 0.71 lower) |  | 296 (2 studies) | ⊕⊕⊝⊝ **low**^1,2^ | SMD -0.95 (-1.19 to -0.71) |
| **sE-selectin - quasi RCT** |  | The mean se-selectin - quasi rct in the intervention groups was **1.4 standard deviations lower** (1.8 to 1 lower) |  | 120 (1 study) | ⊕⊕⊝⊝ **low** | SMD -1.4 (-1.8 to -1) |
| **Von Willebrand factor (****vWF) - RCTs** |  | The mean von willebrand factor (vwf) in the intervention groups was **25.27 lower** (33.15 to 17.39 lower) |  | 470 (4 studies) | ⊕⊕⊝⊝ **low**^1,2^ |  |
| **Activated partial thromboplastin time (APTT) - RCTs** |  | The mean activated partial thromboplastin time (aptt) - rcts in the intervention groups was **4.69 lower** (7.7 to 1.67 lower) |  | 400 (3 studies) | ⊕⊕⊝⊝ **low**^1,2^ |  |
| **Activated partial thromboplastin time (APTT) - quasi RCT** |  | The mean activated partial thromboplastin time (aptt) - quasi rct in the intervention groups was **8.15 lower** (10.44 to 5.86 lower) |  | 82 (1 study) | ⊕⊕⊝⊝ **low** |  |
| **Platelet counts- RCTs** |  | The mean platelet counts in the intervention groups were **49.68 higher** (32.79 to 66.58 higher) |  | 400 (3 studies) | ⊕⊕⊕⊝ **moderate**^2^ |  |
| **D-Dimer- RCTs** |  | The mean d-dimer in the intervention groups was **3.79 lower** (6.51 to 1.07 lower) |  | 400 (3 studies) | ⊕⊕⊝⊝ **low**^1,2^ |  |
| *The basis for the **assumed risk** (e.g., the median control group risk across studies) is provided in footnotes. The **corresponding risk** (and its 95% confidence interval) is based on the assumed risk in the comparison group and the **relative effect** of the intervention (and its 95% CI).  **CI:** Confidence interval; **RR:** Risk ratio; | | | | | | |
| GRADE Working Group grades of evidence **High quality:** Further research is very unlikely to change our confidence in the estimate of effect.  **Moderate quality:** Further research is likely to have an important impact on our confidence in the estimate of effect and may change the estimate. **Low quality:** Further research is very likely to have an important impact on our confidence in the estimate of effect and is likely to change the estimate. **Very low quality:** We are very uncertain about the estimate. | | | | | | |
| ^1^ Random sequence generation and the blinding methods were unclear, and we decided to downgrade the quality of evidence as risk of bias.  ^2^ There was serious heterogeneity among the studies included. Overall, we decided to downgrade by one level.  ^3^ Explanations about why three domains of ROB assessments were not sufficient for downgrading the certainty of evidence:  Incomplete accounting of patients and outcome events, Selective outcome reporting, and Other limitations:  All trials studying SFI for severe pneumonia were conducted in hospital and included inpatients during the treatments, and there was no loss to follow-up and failure to adhere to the intention to treat principle when indicated with complete medical records. Besides, the treatment duration of SFI was based on standard drug instructions, restricted to requirements of medical ethics, and supervised by hospitals, thus, stopping early for benefit observed in randomized trials, in particular in the absence of adequate stopping rules use of unvalidated patient-reported outcomes, and reporting of some outcomes and not others on the basis of the results were nearly impossible. Therefore, the quality of the evidence for theses domains may not be downgraded. | | | | | | |
