# Supplementary material for: Efficacy and Safety of Shenfu Injection for Severe Pneumonia in the Elderly: A Systematic Review and Meta-Analysis Based on Western and Eastern Medicine
Source: Front Pharmacol. 2022 Aug 25;13:779942. doi: 10.3389/fphar.2022.779942 (PMC9454296; doi:10.3389/fphar.2022.779942)
Supplement: Supplementary file 4 [file DataSheet1.docx]

**The references of excluded studies**

18 articles were eliminated for the following reasons: not elderly patients (n=11)(Li, 2005;Li, 2012;Weng and Huang, 2016;Yu et al., 2016;Zhang, 2016;Li et al., 2017;Meng et al., 2018;Zhang, 2018;Zhang and Feng, 2019;Wang et al., 2021;Yang and Wang, 2022), the intervention in the SFI group or control group did not meet the inclusion criteria (n=6)(Li et al., 2011;Xia and Xie, 2017;Liu et al., 2018;Guo et al., 2019;Pan and Chen, 2020;Cao et al., 2021), have inappropriate outcome measures (n=1) (Deng and Wu, 2010).

**REFERENCES**

Cao, Q.C., Wang, G.A., and Wang, G.F. (2021). Effect of Shenfu Injection Combined with Xuebijing Injection on Blood Gas Indicators and Related Stress Hormone Levels in Senile Patients with Severe Pneumonia. *New Chinese Medicine* 53**,** 27-30.

Deng, Y., and Wu, D. (2010). Observation on 50 Cases of Senile Pneumonia Treated with Piperacillin Sodium and Shenfu Injection. *Journal of Practical Traditional Chinese Medicine* 26**,** 840-841.

Guo, Y., Geng, L., Shu, J., Zhang, Y., Xue, X., Liu, H., and Zang, S. (2019). Clinical effect of Tanreqing Injection combined with Shenfu Injection in the treatment of senile severe pneumonia. *China Modern Medicine* 26**,** 151-154.

Li, J., Chen, J., Zhu, T., and Wu, X. (2017). Clinical Observation of Shenfu Injection Combined with Linezolid in the Treatment of Severe Pneumonia Caused by Methicillin-resistant Staphylococcus aureus. *China Pharmacy* 28**,** 2800-2803.

Li, P. (2012). Hemodynamic effects of Shenfu Injection on severe pneumonia. *Guide of China Medicine* 10**,** 52-53.

Li, X. (2005). Clinical Analysis of Treating Infantile Mycoplasma Pneumonia Comibined with Mycoplasmal Damage with Shenfu Zhusheye. *Journal of Henan University of Chinese Medicine* 20**,** 60-61.

Li, Y., Tian, W., Wu, Q., and Zhang, R. (2011). Analysis of the clinical efficacy of integrated traditional Chinese and western medicine in the treatment of 100 elderly patients with severe pneumonia. *Chinese Critical Care Medicine* 23**,** 44-45.

Liu, J., Wu, X., Wang, Y., and Li, B. (2018). Clinical Analysis of Chinese Combined with Western Medicine Treatment for Patients with Senile Severe Pneumon. *Chinese Archives of Traditional Chinese* 36**,** 1458-1461.

Meng, M., Ye, D., Xi, J., and Liu, X. (2018). Retrospective Clinical Study of the Efficacy of Shenfu Injection in the Adjuvant Treatment of Severe Community-acquired Pneumonia. *China Pharmacist* 21**,** 662-665.

Pan, Y., and Chen, Z. (2020). Clinical effect analysis of Tanreqing injection combined with Shenfu injection in the treatment of severe pneumonia in the elderly. *Special Health***,** 42-44.

Wang, Z., Xie, Z., Wang, Z., Liu, C., and Xu, K. (2021). The Clinical Observation of Shenfu injection as an adjuvant for severe pneumonia after thoracic surgery

(Yang Deficiency Syndrome). *Journal of Emergency in Traditional Chinese Medicine* 30**,** 1641-1644.

Weng, X., and Huang, M. (2016). Effects of Shenfu injection on blood lactic acid and prognosis in patients with severe pneumonia. *Harbin Medical Journal* 36**,** 82.

Xia, L., and Xie, Z. (2017). Clinical observation of early application of Shenfu injection combined with Yanhuning injection on elderly patients with severe community － acquired pneumonia and its effect on serum related indexes and coagulation related indexes *Hebei Journal of Traditional Chinese Medicine* 39**,** 1801-1811.

Yang, Y., and Wang, L. (2022). Efficacy of Shenfu Injection Combined with Ceftriaxone in the Treatment of Acute Severe Pneumonia. *China Pharmaceuticals* 31**,** 98-101.

Yu, R., Shen, G., and Huang, B. (2016). Effect of Shenfu injection on serum SOD and MDA in patients with severe pneumonia accompanied by myocardial injury. *Shaanxi Journal of Traditional Chinese Medicine* 37**,** 1336-1337.

Zhang, G. (2018). Effects of the Shenfu injection on blood lactic acid and prognosis in patients with severe pneumonia. *Clinical Journal of Chinese Medicine* 10**,** 72-73.

Zhang, J., and Feng, Q. (2019). The clinical efficacy and mechanism of Shenfu injection combined with azithromycin on infantile mycoplasma pneumonia. *Journal of Xi＇an Jiaotong University(Medical Sciences)* 40**,** 1012-1017.

Zhang, R. (2016). Efficacy of L-carnitine combined with Shenfu injection in the treatment of children with mycoplasma pneumonia complicated with myocardial damage. *Journal of Medical Theory and Practice* 29**,** 1314-1316.
